# Structural myelin defects are associated with low axonal ATP levels but rapid recovery from energy deprivation in a mouse model of spastic paraplegia

**DOI:** 10.1371/journal.pbio.3000943

**Published:** 2020-11-16

**Authors:** Andrea Trevisiol, Kathrin Kusch, Anna M. Steyer, Ingo Gregor, Christos Nardis, Ulrike Winkler, Susanne Köhler, Alejandro Restrepo, Wiebke Möbius, Hauke B. Werner, Klaus-Armin Nave, Johannes Hirrlinger

**Affiliations:** 1 Department of Neurogenetics, Max Planck Institute of Experimental Medicine, Göttingen, Germany; 2 Electron Microscopy Core Facility, Max Planck Institute of Experimental Medicine, Göttingen, Germany; 3 Third Institute of Physics, Georg August University, Göttingen, Germany; 4 Carl Ludwig Institute for Physiology, Faculty of Medicine, University of Leipzig, Leipzig, Germany; Oregon Health & Science University, UNITED STATES

## Abstract

In several neurodegenerative disorders, axonal pathology may originate from impaired oligodendrocyte-to-axon support of energy substrates. We previously established transgenic mice that allow measuring axonal ATP levels in electrically active optic nerves. Here, we utilize this technique to explore axonal ATP dynamics in the *Plp*^null/y^ mouse model of spastic paraplegia. Optic nerves from *Plp*^null/y^ mice exhibited lower and more variable basal axonal ATP levels and reduced compound action potential (CAP) amplitudes, providing a missing link between axonal pathology and a role of oligodendrocytes in brain energy metabolism. Surprisingly, when *Plp*^null/y^ optic nerves are challenged with transient glucose deprivation, both ATP levels and CAP decline slower, but recover faster upon reperfusion of glucose. Structurally, myelin sheaths display an increased frequency of cytosolic channels comprising glucose and monocarboxylate transporters, possibly facilitating accessibility of energy substrates to the axon. These data imply that complex metabolic alterations of the axon–myelin unit contribute to the phenotype of *Plp*^null/y^ mice.

## Abstract 2

Imaging of ATP dynamics in the optic nerve axons of mice lacking the major myelin protein PLP (a model of spastic paraplegia) reveals complex alterations in the metabolic interaction between oligodendrocytes and axons, associated with structural deficits of myelin.

## Introduction

Myelination is a key property of the nervous system of most vertebrate species including humans [1]. Myelin provides insulation of axons and thus allows fast saltatory action potential propagation [2]. However, myelin also prevents direct access of energy substrates to most of the myelinated axon [3]. It has thus been proposed that oligodendrocytes metabolically support axons by providing energy substrates such as lactate [3–8]. However, metabolic support of axons may not suffice in many neurological disorders in which axons degenerate, including the heterogeneous group of hereditary spastic paraplegias [9].

The X-chromosomal *PLP1* gene encodes proteolipid protein (PLP), the most abundant structural protein of central nervous system (CNS) myelin [10–12]. Genetic defects affecting the *PLP1* gene cause the human neurological disorder X-linked spastic paraplegia type 2 (SPG2) [13,14]. In addition to being active in oligodendrocytes, the *PLP1* gene is also active at very low level in CNS neurons [15], yet the neuropathological investigation of mice harboring an oligodendrocyte-specific deletion of the *Plp* gene showed that the progressive degeneration of long-projecting axons is caused by the loss of a PLP-dependent oligodendroglial function [16]. The most commonly used model of SPG2 is *Plp*^null/y^ mice [17,18], in which impaired axonal transport [19] and axonal swellings, as early signs of axonopathy, emerge throughout the CNS by postnatal day 18 [12,18,20]. Yet, clinical signs of disease including motor impairment do not reach significant levels before 16 months of age, when extensive degeneration of long axons in the spinal cord is observed [18]. However, the mechanistic link between genetic defects originally affecting oligodendrocytes and the ultimate degeneration of myelinated axons has remained largely hypothetical.

Considering that oligodendrocytes have been suggested to contribute to axonal energy metabolism by providing lactate to the axon [4,5], we hypothesized that axonal degeneration in *Plp*^null/y^ mice is associated with an early subclinical perturbation of axonal energy metabolism. Indeed, the axonopathy in *Plp*^null/y^ mice [18,21] resembles that in mice with a heterozygous deletion of the lactate transporter MCT1/SLC16A1 [5]. We recently established an experimental system allowing direct assessment of ATP levels in electrically active axons in optic nerves ex vivo [8]. The fluorescent ATP sensor ATeam1.03^YEMK^ [22] was expressed in neurons in the transgenic mouse line ThyAT, allowing confocal imaging of the ATP sensor in optic nerve axons in combination with recordings of compound action potentials (CAPs) [8]. In the present work, we crossbred ThyAT mice with *Plp*^null/y^ mice to test if axonal energy homeostasis is disturbed in this mouse model of SPG2. Indeed, we find that loss of PLP-dependent oligodendroglial function impairs ATP levels in white matter axons. During electrical stimulation of axons, ATP levels show roughly similar dynamics in optic nerves from *Plp*^null/y^ mice as from wild-type mice; however, CAP decrease is more pronounced. Surprisingly, both CAPs and ATP levels declined more slowly during glucose deprivation (GD) and recovered faster after glucose reperfusion in *Plp*^null/y^ compared to control nerves. Myelin of *Plp*^null/y^ mice displays structural defects including increased numbers of cytosolic channels and increased expression of transporters for energy substrates like GLUT1 and MCT1, suggesting enhanced accessibility of energy substrates to the axon. Together, our data show that deletion of PLP, an oligodendroglial protein, results in a complex metabolic imbalance between the axonal and glial compartment, and illustrate to our knowledge for the first time that the ThyAT transgenic model is a useful tool when scrutinizing axonal ATP metabolism in neurological disorders.

## Results and Discussion

**Fig 1:**
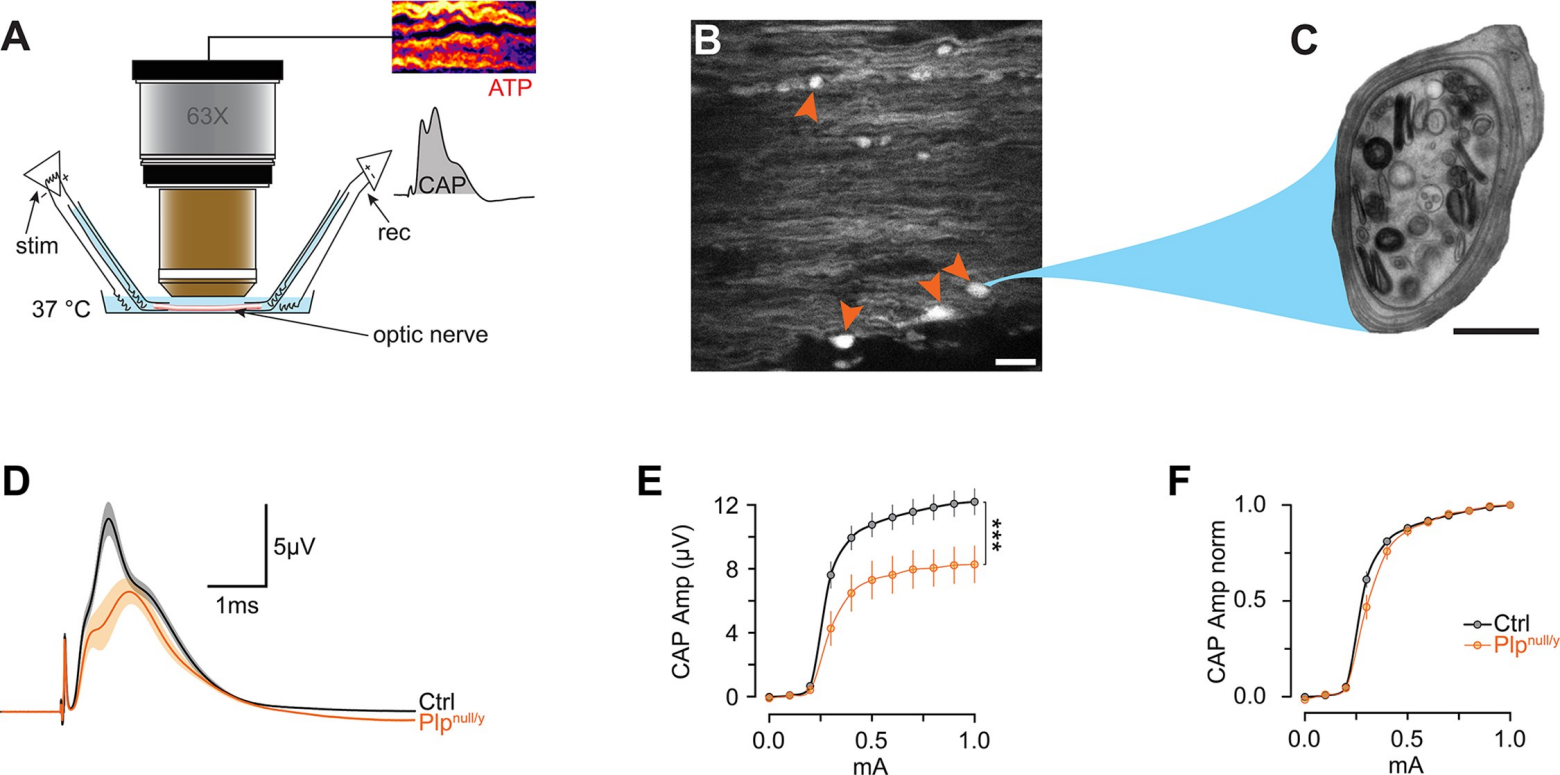
Experimental approach to study ATP homeostasis in the optic nerve of *Plp*^null/y^ mice. *Plp*^null/y^ mice were crossbred with ThyAT mice expressing the fluorescent ATP sensor ATeam1.03^YEMK^ in neurons. (A) Scheme of the experimental setup allowing imaging of the ATP sensor while simultaneously stimulating and recording from optic nerves (modified from [8]). (B) Confocal image of an acutely recorded optic nerve from a ThyAT/*Plp*^null/y^ mouse showing axonal swellings (red arrowheads). Shown is the YFP channel of the ATP sensor. Scale bar: 10 μm. (C) Electron microscopic image of an axonal swelling in an optic nerve axon of a *Plp*^null/y^ mouse. Scale bar: 500 nm. (D) Compound action potential (CAP) of optic nerves of wild-type (control [Ctrl]) and *Plp*^null/y^ mice. Shown is the mean ± SEM of *n* = 10 and 8 optic nerves from *N* = 10 and 8 mice for wild type and *Plp*^null/y^, respectively. (E) Excitability of optic nerve measured at different stimulus intensities (0–1 mA). Shown is the mean ± SEM of *n* = 10 and 8 optic nerves from *N* = 10 and 8 mice for Ctrl and *Plp*^null/y^, respectively. ***p < 0.001, Student *t* test. (F) Same data as in (E), but normalized to the maximal CAP. Data underlying this figure can be found in S1 Data.

To study the dynamics of ATP in electrically active axons in *Plp*^null/y^ mice [18,19], these mice were crossbred to the ThyAT mouse line expressing the ATP sensor ATeam1.03^YEMK^ in neurons. Optic nerves were investigated by combining confocal imaging of the ATP sensor with simultaneous recording of CAPs (Fig 1A) [8]. At the age of 10 weeks, acute confocal imaging of the axoplasmic ATP sensor displayed occasional axonal swellings in *Plp*^null/y^ mice (Fig 1B), which most likely correspond to axonal swellings with accumulation of cell organelles as observed by electron microcopy (Fig 1C). When optic nerves were electrically stimulated, the observed amplitudes of the CAP were significantly smaller in *Plp*^null/y^ mice compared to *Plp*^wt/y^ littermate controls (Fig 1D and 1E). However, normalizing CAPs revealed that the excitability of the axons is similar in optic nerves of both *Plp*^null/y^ and control mice (Fig 1F). This implies that in *Plp*^null/y^ mice fewer active axons contribute to nerve conduction, yet these axons display similar excitability.

**Fig 2:**
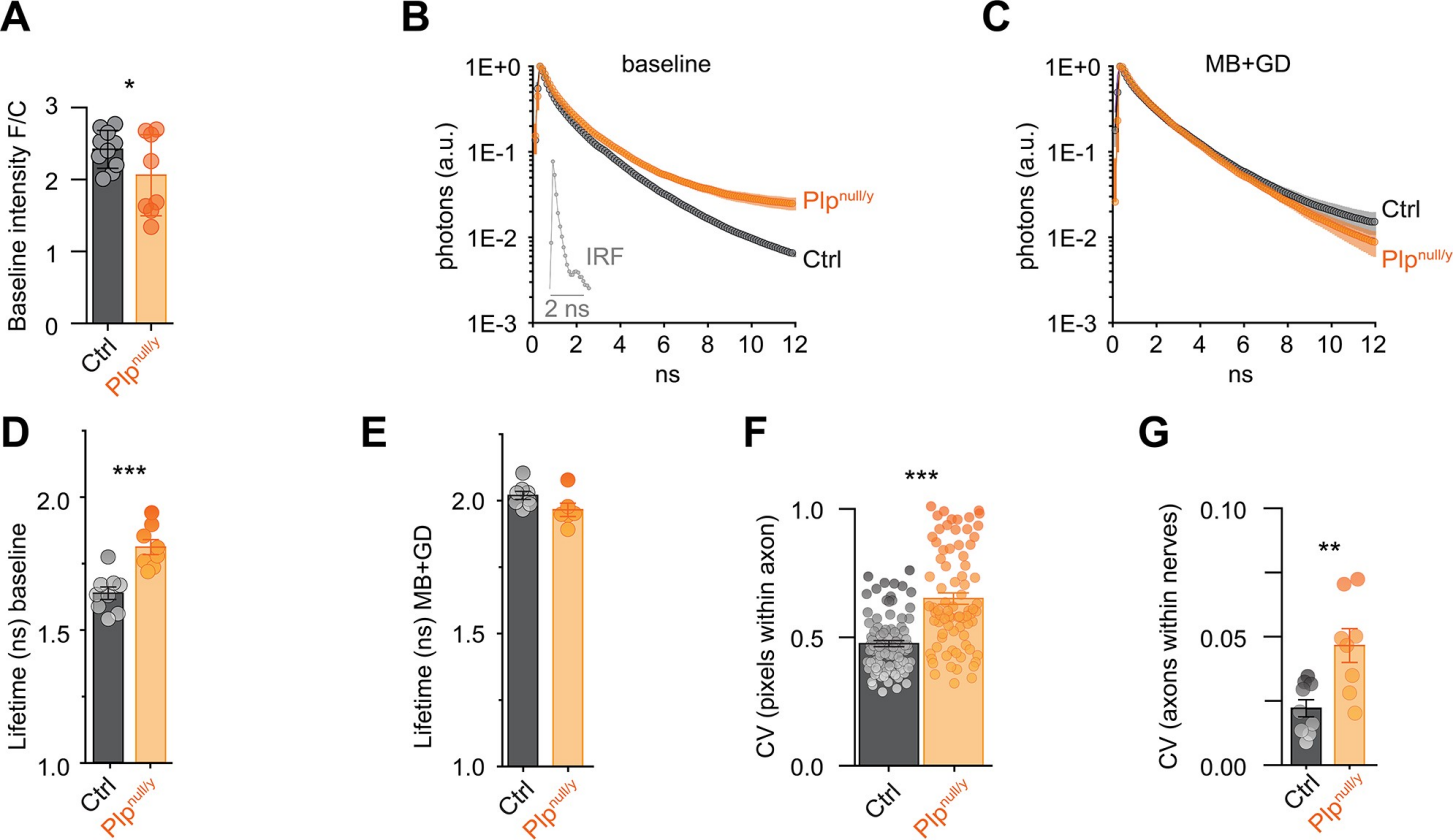
Axoplasmic ATP concentration is reduced in *Plp*^null/y^ mice under basal conditions. (A) ATP sensor ratio at baseline conditions. *n* = 10 and *n* = 8 nerves from *N* = 10 and 8 mice (age 10 weeks) for ThyAT/*Plp*^wt/y^ (control [Ctrl]) and ThyAT/*Plp*^null/y^ (*Plp*^null/y^), respectively. (B) Fluorescence decay of the donor fluorophore of the ATP sensor in optic nerve axons of Ctrl and *Plp*^null/y^ mice under baseline incubation conditions. A slower decay and, consequently, an increase of the fluorescence lifetime correspond to a decrease in ATP concentration. The inset shows the instrument response function (IRF). Shading represents SEM. (C) Average fluorescence decay of the same axons as in (B) but after depletion of ATP by mitochondrial blockage and glucose deprivation (MB+GD). (D) Quantification of the fluorescence lifetime $\bar{\tau }$ (ns) of the ATP sensor in axons under baseline conditions, indicating a lower ATP concentration in *Plp*^null/y^ nerves. (E) Quantification of the fluorescence lifetime $\bar{\tau }$ (ns) of the ATP sensor in axons exposed to MB+GD, indicating that the longest fluorescent lifetime is obtained for each nerve under this energy-depriving condition. The average fluorescence decay of *n* = 6–9 nerves from *N* = 4–5 mice (10 weeks old) is shown in (B–E). (F) The variability of the ATP concentration within individual axons was analyzed by calculating the coefficient of variation (CV) of the fluorescence lifetime of all pixels within an axon; 88 and 79 axons from *n* = 9 and 8 nerves from *N* = 5 and 4 animals were analyzed for control and *Plp*^null/y^ nerves, respectively. (G) CV of the ATP sensor fluorescence lifetime of axons within a nerve. 8 to 10 axons each from *n* = 9 and 8 nerves from *N* = 5 and 4 animals were included in the analysis for control and *Plp*^null/y^ nerves, respectively. *p < 0.05, **p < 0.01, ***p < 0.001; Student *t* test (A, D, E, G), Welch’s test (F). Data underlying this figure can be found in S1 Data.

A moderately reduced ATP sensor signal was observed by confocal imaging in the optic nerve of *Plp*^null/y^ mice compared to controls under baseline conditions (Fig 2A), suggesting a lower basal concentration of ATP in axons. To further substantiate this notion, 2-photon laser scanning fluorescence lifetime imaging (FLIM) was employed (Fig 2B–2G; S1 Fig). In HEK293 cells patched with pipette solutions containing different ATP concentrations, the fluorescence lifetime of the ATP sensor showed a direct dependency on the concentration of ATP (S2 Fig). Unfortunately, the absolute values of the lifetime were slightly different from the values obtained in axons of the optic nerves (most likely because of the different cellular environment), thereby precluding a direct calculation of the ATP concentration within the axon. The ATP sensor in optic nerve axons of *Plp*^null/y^ mice showed an increased fluorescence lifetime compared to control mice under basal conditions, supporting a reduced ATP concentration (Fig 2B and 2D; S3 Fig). As a control, nerves were depleted of ATP by blocking mitochondria using azide and depriving the nerves of glucose, thereby fully inhibiting ATP synthesis (referred to as MB+GD [8]). This treatment further increased fluorescent lifetime to a similar value in both control and *Plp*^null/y^ optic nerves (Fig 2C and 2E; S3 Fig), supporting the validity of the lifetime measurements. To exclude a contribution of background fluorescence to the fluorescence lifetime measurements of the ATP sensor, tissue autofluorescence was assessed in nerves from wild-type mice lacking expression of the ATP sensor. Using the same imaging conditions, no signal and no decay were observed in these optic nerves (S4 Fig), thus excluding a contribution of tissue autofluorescence. Furthermore, the pH dependency of the fluorescence lifetime of the ATP sensor was analyzed in HEK293 cells taking advantage of the ATP-binding-deficient sensor variant AT1.03^R122K/R126K^ [22] to exclude lifetime changes caused by alterations of the ATP concentration due to active, energy-consuming pH regulation of the cells. The ATP sensor fluorescence lifetime slightly increased with increasing pH (S4 Fig). However, this pH-induced change in lifetime does not invalidate the conclusions of different ATP levels in control and *Plp*^null/y^ optic nerves because the change in lifetime induced by a rather large pH change is small compared to the difference observed between control and *Plp*^null/y^ mice. We also note that tissues with suboptimal energy production mostly become more acidic [23–25], including the optic nerve [26]. Indeed, such a decrease of pH in optic nerves from *Plp*^null/y^ mice would result in a decrease of the fluorescence lifetime of the ATP sensor; however, an increased lifetime was observed in *Plp*^null/y^ mice (Fig 2B and 2D). Therefore, a potential impact of pH on the lifetime measurement would, if anything, most likely result in an underestimation of the difference in ATP concentration between control and *Plp*^null/y^ axons. Together, these findings indicate that *Plp*^null/y^ mice display reduced axonal ATP levels.

To address the variability of the axonal ATP concentration, the coefficient of variation (CV) of the lifetime of the ATP sensor across single pixels of individual axons (Fig 2F), as well as across axons within single nerves (Fig 2G), was analyzed. Importantly, these analyses revealed the same average lifetime as the analysis of the whole optic nerve (S5 Fig). The CV of the fluorescence lifetime recorded in the different pixels within individual axons was significantly larger in *Plp*^null/y^ optic nerves compared to control optic nerves (Fig 2F), indicating a larger variability of the intra-axonal ATP concentration. The variation of the ATP concentration of individual axons within each nerve was also larger in *Plp*^null/y^ optic nerves (Fig 2G). Moreover, in female *Plp*^null/wt^ mice (i.e., female mice heterozygous for the *Plp*^null^ allele), each oligodendrocyte inactivates either the wild-type or the mutant allele of the X-chromosomal *Plp* gene, leading to a mosaic cellular pattern of expression and lack of PLP. Consistently, an intermediate basal ATP concentration as well as an intermediate CV was observed in these heterozygous *Plp*^null/wt^ mice (S6 Fig). Together, the overall lower ATP levels in conjunction with their increased variability in optic nerve axons of *Plp*^null/y^ mice support the concept that axonal energy metabolism depends on oligodendroglial support [4–8,27].

The emergence of pathological axonal swellings in *Plp*^null/y^ mice has been attributed to a “traffic jam” of axonal transport [19], which may plausibly be attributed to the present observation of an overall reduced ATP in *Plp*^null/y^ axons. Of note, axonal swellings in the optic nerves of *Plp*^null/y^ mice displayed either a shorter or longer fluorescence lifetime of the ATP sensor compared to neighboring axons (S1 Fig). This observation implies that axonal swellings are heterogeneous with respect to their ATP concentration, suggesting that axonal swellings are not (only) the consequence of a local lack of ATP within the axon. In case other, yet unidentified, mechanisms cause the accumulation of organelles including mitochondria within these swellings, the local accumulation of mitochondria might result in increased local ATP production and thus increased ATP concentration within the swellings. Indeed, extensive 3D electron microscopic analysis revealed that most swellings contain a mixture of organelles, but 16% of all axonal swellings were almost exclusively composed of mitochondria [20]. These mitochondria will also not be available for ATP production in adjacent axonal segments, possibly leading to a reduced ATP concentration. Yet, we note that we cannot formally exclude artificial changes of lifetime, e.g., owing to unidentified molecular interactions of the sensor within these swellings.

To further assess the ATP dynamics in axons actually propagating action potentials, confocal microscopy was used to monitor the ATP sensor signal. This ATP sensor signal based on fluorescence intensity ratio is barely affected by intracellular pH in the physiological pH range [8,22,28,29], and tissue autofluorescence only contributes a minor and invariant part to the observed signal (S4 Fig). Furthermore, simultaneous recording of the ATP-independent fluorescence in the YFP channel revealed no overt differences between control and *Plp*^null/y^ optic nerves (as exemplified for the harsh metabolic challenge of MB+GD; S4 Fig), suggesting that these treatments do not induce large differences of pH between genotypes. Therefore, the changes in the ATP sensor signal reflect a corresponding dynamic change in the ATP concentration within the axoplasm.

**Fig 3:**
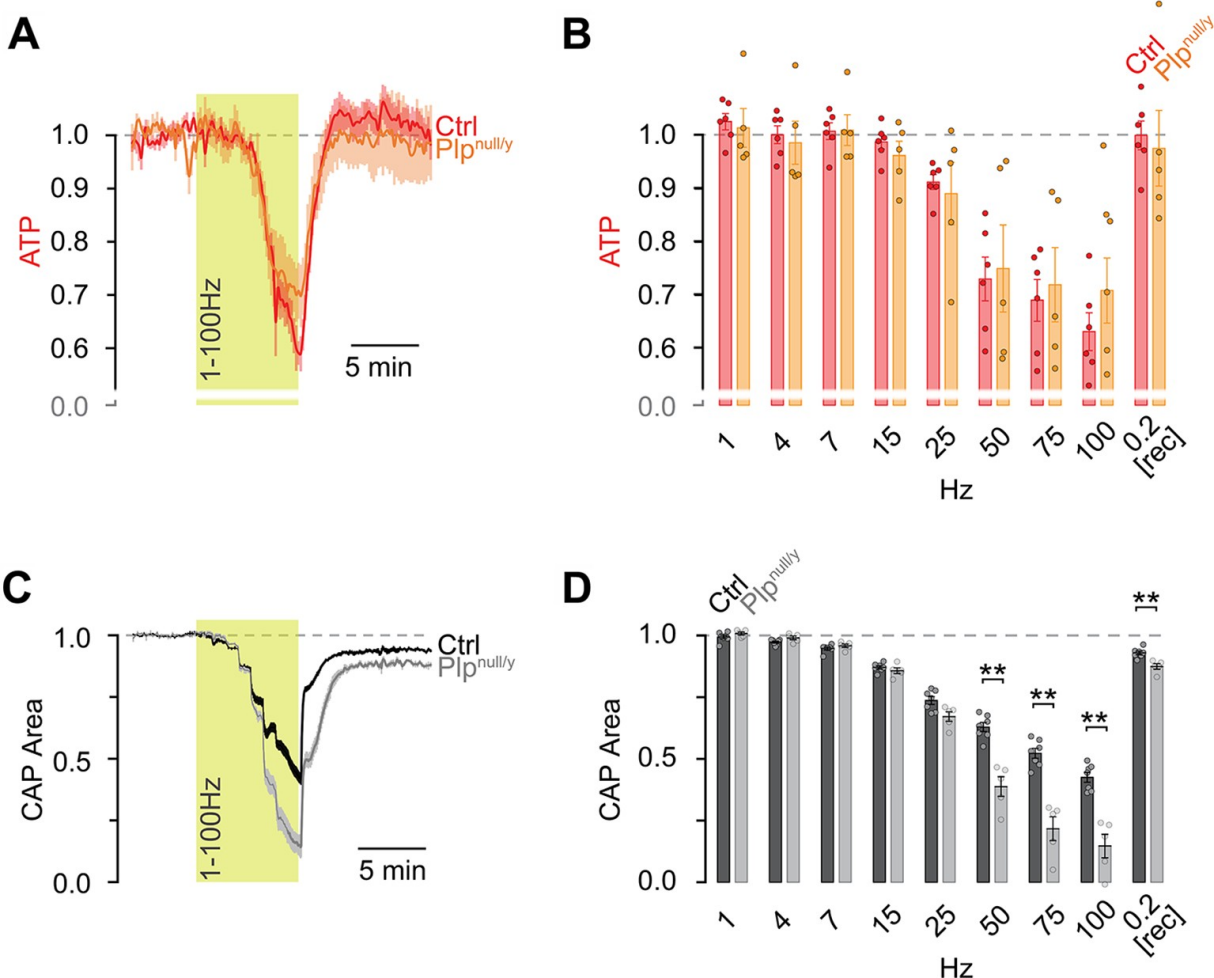
During electrical stimulation the decrease in compound action potential (CAP), but not of ATP, is larger in *Plp*^null/y^ mice. Optic nerves from control (Ctrl) or *Plp*^null/y^ mice were challenged by electrical stimulation increasing every 30 s stepwise from 1 Hz to 100 Hz. (A) Time course of the ATP sensor signal normalized to baseline (set as 1) and MB+GD (mitochondrial blockage and glucose deprivation; set as 0). (B) Quantification of the ATP sensor signal for the last 15 s during each stimulation with the indicated frequencies. Same nerves as in (A). (C) Dynamics of CAPs normalized to baseline (set as 1) and MB+GD (set as 0). (D) Quantification of the CAPs for the last 15 s during each stimulation with the indicated frequencies. Same nerves as in (C). Shown is the mean ± SEM. Dots in (B) and (D) indicate data points from single nerves; *n* = 6 (ATP Ctrl), 7 (CAP Ctrl), and 5 (ATP/CAP *Plp*^null/y^) nerves from *N* = 6, 7, and 5 mice, respectively. **p < 0.01; Student *t* test (B and D). Data underlying this figure can be found in S1 Data.

Optic nerves from ThyAT/*Plp*^null/y^ mice were functionally challenged by electrical stimulation using a “ramp” protocol with progressively increasing stimulation frequency ranging from 1 Hz to 100 Hz (each frequency was maintained for 30 s; Fig 3). During stimulation, the ATP sensor signal dropped gradually with increasing stimulation frequency; however, no difference was observed between control and *Plp*^null/y^ optic nerves (Fig 3A and 3B). After cessation of stimulation, the ATP sensor signal recovered to baseline levels in both control and *Plp*^null/y^ axons. In contrast, CAPs dropped in a stepwise fashion with increasing stimulation frequency (Fig 3C), and the amplitude of this decrease was larger in *Plp*^null/y^ mice compared to controls (Fig 3D). Furthermore, CAPs in control nerves recovered quickly and almost completely after the stimulation, while in *Plp*^null/y^ mice recovery was moderately but significantly lower (Fig 3D). The close correlation of the decrease in ATP and CAP in optic nerves from control mice confirms our previous observation with a different stimulation paradigm [8]. Strikingly, both pharmacologically interfering with metabolism [8] and genetic deletion of *Plp* break this close correlation, but in opposite directions. When metabolism is pharmacologically perturbed, CAPs remain similar to those of undisturbed nerves but the decrease in ATP is enhanced, indicative of reduced ATP production under conditions of similar consumption [8]. In contrast, ATP dynamics in *Plp*^null/y^ mice show no significant difference compared to controls, but the decrease of action potential conduction is significantly exacerbated. As changes in both ATP sensor signal and CAPs refer to changes relative to their baseline levels, which are lower in *Plp*^null/y^ mice, the interpretation needs some caution. Yet, these data imply that either ATP consumption is not increased in *Plp*^null/y^ compared to control optic nerves during high frequency stimulation or ATP production can fully compensate for an increased demand also in *Plp*^null/y^ mice. The enhanced decrease in CAP is thus likely not a direct consequence of an energy deficit but may rather be induced by a disturbance of ion homeostasis leading to increased accumulation of K^+^ in the extracellular space during high frequency stimulation [30].

**Fig 4:**
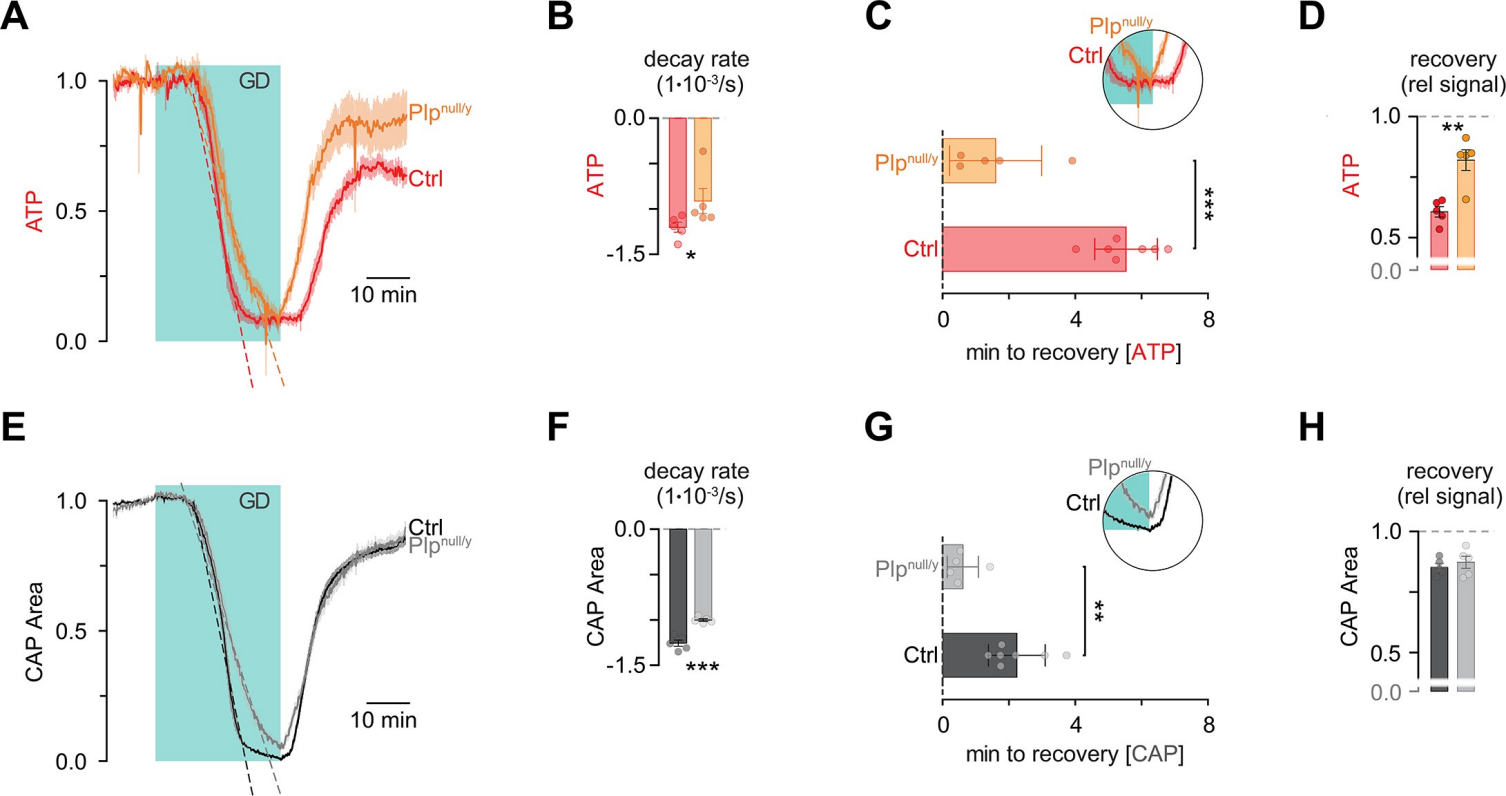
ATP levels in optic nerves from *Plp*^null/y^ mice recover faster and more completely from glucose deprivation (GD). Optic nerves from *Plp*^wt/y^ (control [Ctrl]) or *Plp*^null/y^ mice were challenged by GD for 30 min (indicated by the light blue box). Afterwards, glucose (10 mM) was reperfused for an additional 45 min. (A) Time course of the ATP sensor signal in Ctrl and *Plp*^null/y^ mice. (B) Rate of ATP sensor signal decay. (C) Delay time of the onset of recovery of the ATP sensor signal after the start of reperfusion with glucose. (D) Amplitude of recovery of the ATP sensor signal after reperfusion with glucose. (E) Time course of compound action potentials (CAPs) in Ctrl and *Plp*^null/y^ mice. (F) Rate of CAP decay. (G) Delay time of the onset of recovery of CAP after the start of reperfusion with glucose. (H) Amplitude of recovery of CAP after reperfusion with glucose. *n* = 5 (Ctrl; B, D, F, H), 7 (Ctrl; C, G), and 5 (*Plp*^null/y^) optic nerves from *N* = 5, 7, and 5 mice, respectively. *p < 0.05, **p < 0.01, ***p < 0.001; Student *t* test. Data underlying this figure can be found in S1 Data.

To assess the metabolic properties of *Plp*^null/y^ optic nerves during limitation of energy substrate supply, we next challenged these white matter tracts by GD (Fig 4). Strikingly, ATP levels in *Plp*^null/y^ nerves dropped more slowly compared to control nerves (Fig 4A and 4B). During reperfusion with glucose, control nerves displayed a lag time for the recovery of ATP as previously observed [8]. Unexpectedly, however, the onset of recovery of axonal ATP levels was significantly earlier in *Plp*^null/y^ mice (Fig 4A and 4C). Finally, while the ATP sensor signal in control nerves recovered only to 60.7% ± 1.9% of its baseline level, recovery in *Plp*^null/y^ optic nerves reached 82.0% ± 3.8% (Fig 4D). Similar to ATP levels, CAPs decayed more slowly during GD in *Plp*^null/y^ mice compared to controls (Fig 4E and 4F) and began to recover earlier after reperfusion (Fig 4E and 4G). We note that the extent of recovery of CAPs did not significantly differ between control and *Plp*^null/y^ optic nerves (Fig 4H). Together, these experiments indicate that, while having lower basal ATP levels, axons in optic nerves that lack PLP from CNS myelin are able to handle GD better than nerves from control animals.

**Fig 5:**
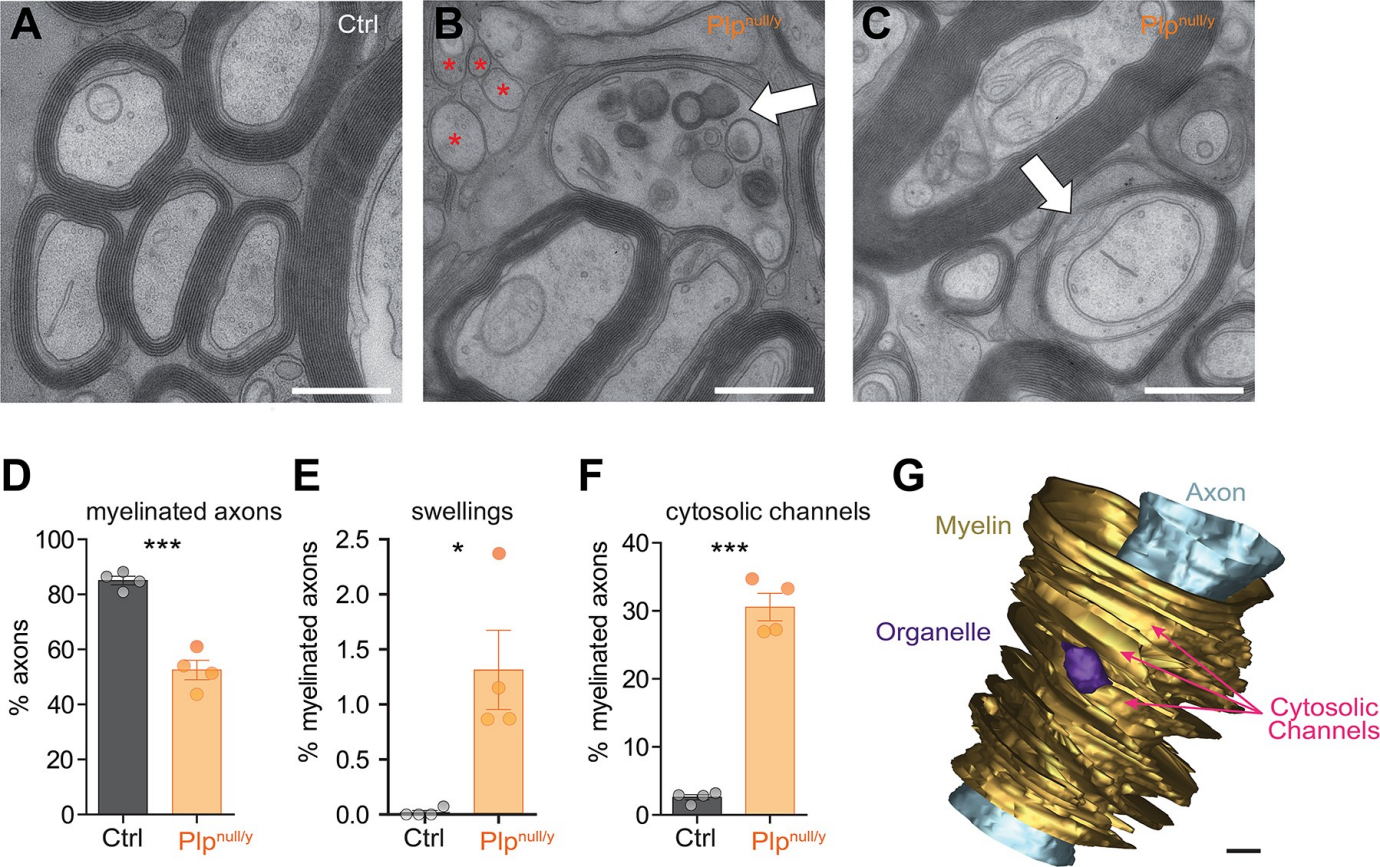
Structure of myelin in *Plp*^null/y^ optic nerves. (A–C) Electron micrographs of high-pressure frozen optic nerves of a 10-week-old control (Ctrl) (A) and *Plp*^null/y^ (B and C) mouse. Asterisks indicate unmyelinated axons; white arrows highlight an axonal spheroid (B) and cytosolic channels (C). The scale bars correspond to 500 nm. (D) Genotype-dependent quantification of the number of myelinated axons in optic nerves dissected from Ctrl and *Plp*^null/y^ mice; 100% refers to all axons. (E) Genotype-dependent quantification of the percentage of axon–myelin units comprising axonal swellings; 100% refers to all myelinated axons.(F) Genotype-dependent quantification of the percentage of axon–myelin units with myelin containing cytosolic channels; 100% refers to all myelinated axons. In (D–F), *n* = 4 and 4 nerves from *N* = 4 and 4 mice for Ctrl and *Plp*^null/y^, respectively. *p < 0.05, ***p < 0.001; Student *t* test. (G) 3D rendering of the structure of an axonal segment and the inner layers of the myelin sheaths obtained using focused ion beam scanning electron microscopy (FIB-SEM). For simplicity, the outermost layer of myelin is not visualized in the 3D reconstruction. Cytosolic channels are colored yellow, the axon is in light blue, and organelle-like structures are in purple. The scale bar corresponds to 200 nm. See S1 Movie for an animated visualization of this 3D structure. Data underlying this figure can be found in S1 Data.

While myelin electrically isolates axons to accelerate action potential propagation, it also shields axons from access to substrates necessary for energy metabolism [3,6,27]. It has been proposed that oligodendrocytes metabolically support axons, thereby bypassing this diffusion barrier [3–7]. We therefore hypothesized that myelin in *Plp*^null/y^ mice may allow more efficient diffusion of energy substrates. To test this idea, we assessed the ultrastructure of myelin in high-pressure frozen optic nerves of *Plp*^null/y^ mice at the age of 75 days (Fig 5), the same age as used for ATP imaging and electrophysiology. As expected, myelin appeared regularly layered in control optic nerves; only a few unmyelinated axons or morphologically abnormal axon–myelin units were observed (Fig 5A and 5D–5F). Conversely, optic nerves of *Plp*^null/y^ mice comprised a larger frequency of unmyelinated axons and of axon–myelin units displaying axonal swellings or cytosolic channels, identified as cytoplasm-filled lamellae splittings in between otherwise compact myelin layers (Fig 5B–5F). These data confirm previous reports of an increased frequency of unmyelinated axons [31,32] and axonal swellings [18,32] in the CNS of *Plp*^null/y^ mice and provide quantification of previous observations of a strongly increased number of cytosolic channels through CNS myelin [33,34]. To visualize a cytosolic channel through myelin 3-dimensionally, an axon–myelin unit of an optic nerve dissected from a *Plp*^null/y^ mouse was reconstructed based on images acquired by focused ion beam scanning electron microscopy (FIB-SEM; Fig 5G; S1 Movie). The reconstruction illustrates 1 example of a myelin sheath radially split to form a cytosolic channel large enough to contain cytoplasm and organelles (Fig 5G). Cytosolic channels through CNS myelin are present during an early stage of normal developmental myelination but largely close by the age of 3 weeks in optic nerves of wild-type mice [35]. Deletion of the *Plp* gene in adult mice after closure of the developmental cytosolic channels results in the late-onset formation of new cytosolic channels as part of the pathology, indicating that also the maintenance of fully compacted myelin free of cytosolic channels depends on continuous expression of PLP [36]. Clarifying whether the presence of cytosolic channels in the myelin of *Plp*^null/y^ mice is caused by impaired closure of developmental or secondary emergence of pathological cytosolic channels will require further 3D electron microscopy analysis with high temporal resolution during development [20]. Yet, we hypothesize that these cytosolic channels may serve as additional diffusion routes for metabolites through the myelin sheaths when PLP is lacking.

**Fig 6:**
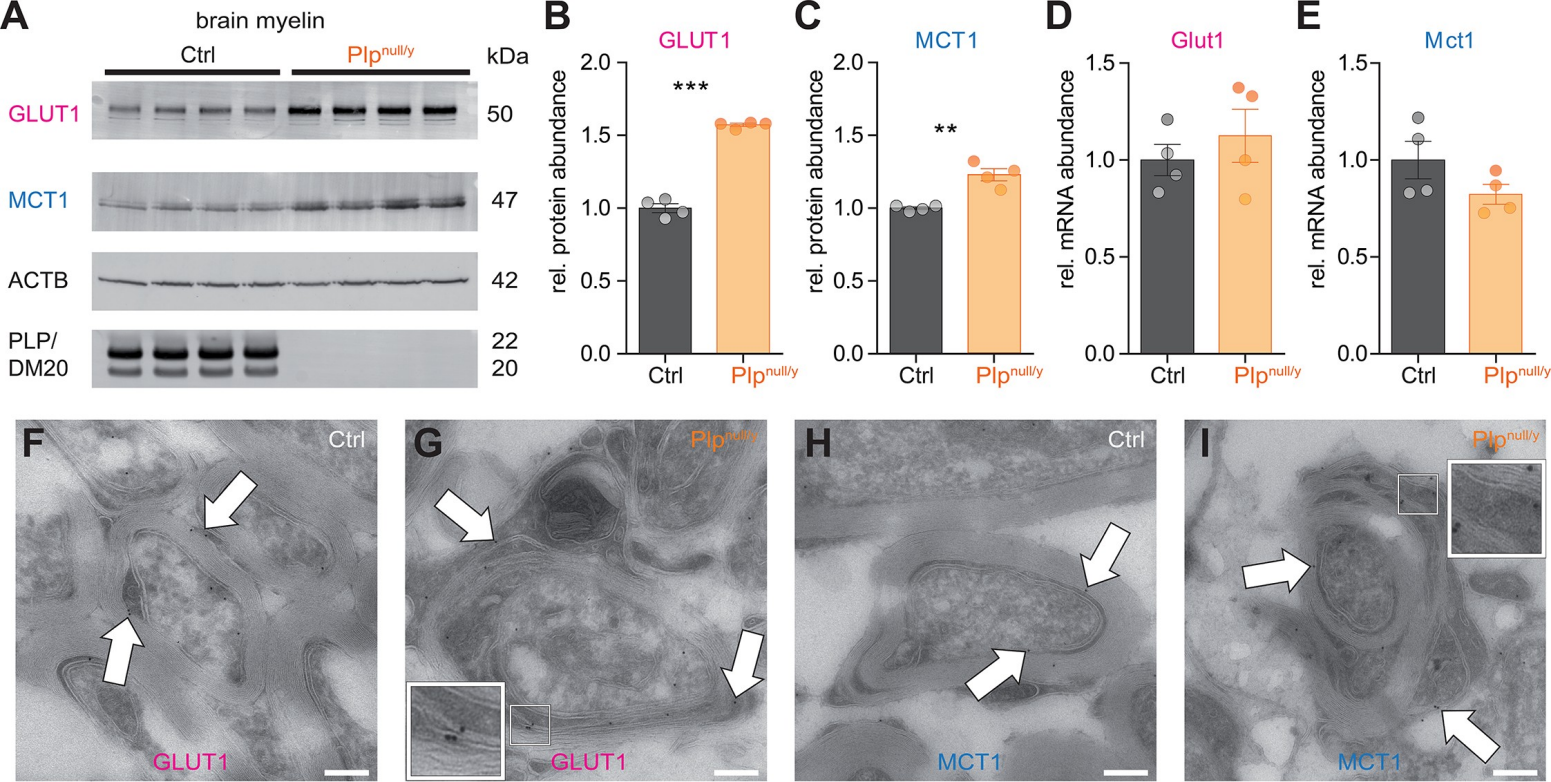
Abundance and localization of GLUT1 and MCT1 in myelin. (A) Immunoblot analysis of myelin purified from the brains of wild-type (control [Ctrl]) and *Plp*^null/y^ mice probed for glucose transporter 1 (GLUT1) and monocarboxylate transporter 1 (MCT1). β-actin (ACTB) was detected as a loading control; PLP/DM20 was detected as genotype control. Blot represents *N* = 4 individual mice for each genotype. (B and C) Genotype-dependent quantification of the immunoblots for GLUT1 (B) and MCT1 (C). The abundance of both proteins is significantly increased in myelin purified from *Plp*^null/y^ brains (*N* = 4 mice for both genotypes). (D and E) Quantitative RT-PCR analysis of the abundance of *Glut1* (D) and *Mct1* (E) mRNAs in the corpus callosum dissected from wild-type (Ctrl) and *Plp*^null/y^ mice (*N* = 4 mice for both genotypes). In (B–E): **p < 0.01, ***p < 0.001; Student *t* test. (F–I) Cryo-immuno electron microscopy to assess the localization of GLUT1 (F and G) and MCT1 (H and I) protein in the myelin of optic nerves dissected from wild-type (F and H) and *Plp*^null/y^ (G and I) mice. White arrows point at gold particles indicating localization of the respective proteins. The scale bars in (F–I) correspond to 200 nm. White boxes are 2.6× zoomed-in magnification of the corresponding box highlighting the gold particles. Original, uncropped Western blots are available in S1 Raw Images. Data underlying this figure can be found in S1 Data.

Considering their relevance for the transmembrane transport of metabolites, we then assessed the abundance of the major glucose and lactate transporters GLUT1 (SLC2A1) and MCT1 (SLC16A1) in myelin purified from the brains of control and *Plp*^null/y^ mice (Fig 6). Indeed, the abundance of both GLUT1 and MCT1 was increased in PLP-deficient myelin according to immunoblot (Fig 6A–6C), in agreement with the hypothesis that PLP-deficient myelin has adopted additional capacity for transporting energy substrates. Notably, by quantitative RT-PCR the abundance of the mRNAs encoding both transporters was unchanged in corpus callosum (Fig 6D and 6E), suggesting that the increased GLUT1 and MCT1 protein abundance occurs post-transcriptionally.

To test whether GLUT1 and MCT1 are associated with cytosolic channels through myelin, their localization in myelin was studied using cryo-immuno electron microscopy of optic nerves. In both control and *Plp*^null/y^ mice, gold particles coupled to antibodies directed against either of these metabolic carriers localized to the inner and outer tongues of myelin (Fig 6F–6I). In addition, in *Plp*^null/y^ mice, labeling of both transporters was also found on the myelin membranes flanking the cytosolic channels (Fig 6G and 6I). The increased abundance of these metabolite transporters in PLP-deficient myelin and their localization to non-compacted myelin subcompartments is in agreement with the hypothesis of enhanced nutrient transport through myelin towards the axon.

In summary, we report the first utilization, to our knowledge, of transgenic ThyAT mice [8] to assess axonal ATP metabolism in a genetic mouse model of an axonopathy caused by a primary impairment of myelin. Imaging axonal ATP is a sensitive and sub-threshold analysis tool for real-time monitoring of metabolic glia–axon interactions in electrically active fiber tracts and provides a readily accessible approximation for axonal metabolism and function under physiological and pathophysiological conditions. We find that *Plp*^null/y^ mice display (i) reduced axonal ATP levels and CAPs and (ii) increased variability of the ATP concentration within single axons but also between different axons, both probably reflecting an early stage of the functional impairments ultimately resulting in the emergence of the histopathological hallmarks of *Plp*^null/y^ mice [18,19]. These lower ATP levels as well as the increased variability of ATP are consistent with the concept that axonal energy metabolism depends on oligodendroglial support [4–8,27]. Alternatively, substantially increased ATP demand by axons in the optic nerve of *Plp*^null/y^ mice (e.g., induced by the structural deficits of myelin; Fig 5), limited by a finite capacity to generate ATP, would also result in lower ATP levels. Indeed, as proper myelination reduces the energy demand of axons, it is conceivable that axons in *Plp*^null/y^ mice have a higher energy demand during action potential propagation. However, lower ATP levels are observed even in unstimulated *Plp*^null/y^ nerves in the absence of electrical activity (Fig 2), but their relative ATP decline during high frequency stimulation is not different compared to controls (Fig 3). This suggests that the capacity of ATP generation is not limiting under basal conditions and that axons in optic nerves of *Plp*^null/y^ mice can cope with increased ATP demand during electrical activity.

Furthermore, we unexpectedly find that during GD, axonal ATP levels decline more slowly and recovery after glucose reperfusion starts earlier in optic nerves from *Plp*^null/y^ mice compared to wild-type mice, indicating that ATP synthesis is even increased in *Plp*^null/y^ axons in the optic nerve under these conditions. Astrocytes contribute to maintaining energy homeostasis within the optic nerve by providing glycogen-derived lactate to the axon [37–39]. The latency of the onset of CAP failure during GD directly correlates with glycogen content of the optic nerve [37]. Astrogliosis indicated by upregulation of GFAP was reported in the corpus callosum and retina of *Plp*^null/y^ mice at 2.5 months of age [12]. In the optic nerve, the number of non-oligodendrocyte cells, presumably astrocytes, was increased at postnatal day 120, but not yet at postnatal day 60, in *Plp*^null/y^ mice [31]. Therefore, it is conceivable that activated astrocytes contribute to the observed phenotype, possibly reflected by the slower decrease of ATP levels and CAPs during GD. In contrast, as astrocytic glycogen is depleted in the mouse optic nerve within 20 min during GD [37], this astrocytic energy reservoir cannot contribute to the observed fast recovery after glucose reperfusion.

Based on these findings we propose the following model: Impairment of oligodendrocyte function due to PLP deficiency results in at least partially unmyelinated axons, which is reflected by their increased frequency observed in cross-sections (Fig 5D) [31,32]. These unmyelinated stretches of axons lack local metabolic support by oligodendrocytes, resulting in locally impaired ATP synthesis and maintenance. As other parts of the axons are still metabolically supported by myelin, this will result in the observed higher variability of ATP levels as well as an on average reduced axonal ATP concentration (Fig 2). In the attempt to compensate, remaining oligodendrocytes upregulate metabolite transporters to increase the metabolic support of axons. Furthermore, while we cannot discriminate whether the occurrence of the increased number of cytosolic channels is a primary consequence of PLP deficiency or whether this is part of a compensatory reaction of functional oligodendrocytes, these channels complement the upregulation of metabolite transporters by providing additional diffusion routes for molecules through myelin. Consistently, the observed rapid recovery from GD in *Plp*^null/y^ mice can be plausibly explained by facilitated diffusion, i.e., an increased diffusion coefficient *D* in Fick’s law: *J* = −*D* * Δ*c*. An increase in *D* will result in the same flux *J* of energy substrates towards the axons at a lower concentration gradient Δ*c*. Because glucose concentration in the tissue increases gradually upon its reperfusion, an increase of *D* would allow a flux of energy substrates sufficient for maintaining axonal ATP earlier during reperfusion at lower Δ*c*. Indeed, this concept is consistent with our experimental findings in optic nerves of *Plp*^null/y^ mice. Small molecules like lucifer yellow diffuse from oligodendrocyte cell bodies towards the non-compacted cytosolic compartments within myelin [40], and it has been suggested that cytosolic channels through otherwise compacted myelin may constitute transport routes for energy metabolites [16,41]. We here extend these findings by providing physiological, although indirect, evidence of increased diffusion of energy substrates in the optic nerves of *Plp*^null/y^ mice. Furthermore, PLP deficiency interferes with the physiological metabolic support by oligodendrocytes to axons in white matter, inducing complex alterations of axonal ATP homeostasis.

## Materials and methods

### Ethics statement

Animal experiments were performed in accordance with the guidelines for the welfare of experimental animals issued by the European Communities Council Directive 2010/63/EU, with the German Animal Welfare Act (Tierschutzgesetz), and with the animal experiment guidelines of the Max Planck Institute of Experimental Medicine. Experiments were approved by the institute’s animal welfare officer and the Landesamt für Verbraucherschutz und Lebensmittelsicherheit (LAVES), the responsible governmental authority of the German federal state of Niedersachsen.

### Transgenic mice

The ThyAT mouse line expressing ATeam1.03^YEMK^ under control of the neuronal *Thy1* promotor (official name: B6-Tg(Thy1.2-ATeam1.03YEMK)AJhi; MGI: 5882597) has been described recently [8]. C57Bl6/N-*Plp*^null/y^ (*Plp*^null/y^) mice [17] were crossbred to ThyAT mice. Mice were bred in the animal facility of the Max Planck Institute of Experimental Medicine, housed in a 12-h/12-h light dark cycle with access to food and water ad libitum and 2–5 mice per cage. For experiments shown in Figs 1–4, control (Ctrl) refers to ThyAT^tg^/*Plp*^wt/y^ mice (i.e., mice wild-type for *Plp*, but expressing the ATP sensor), while *Plp*^null/y^ refers to ThyAT^tg^/*Plp*^null/y^ mice (i.e., mice with deletion of *Plp*, and expressing the ATP sensor). Female *Plp*^null/wt^ mice (i.e., female mice heterozygous for the *Plp*^null^ allele) were used for experiments shown in S6 Fig. Mice were used at 8–12 weeks of age. Mice used for experiments in Figs 5 and 6 did not harbor the ThyAT transgene and were used at postnatal day 75.

### Optic nerve preparation and electrophysiological recordings

Optic nerve preparation and electrophysiological recordings were done as described before [7,8,42]. Optic nerves were excised from decapitated mice, placed into an interface perfusion chamber (Harvard Apparatus) and continuously superfused with artificial cerebrospinal fluid (aCSF) [8]. The perfusion chamber was continuously aerated by humidified carbogen (95% O_2_/5% CO_2_) and experiments were performed at 37°C. Custom-made suction electrodes back-filled with aCSF were used for stimulation and recording as described [7,43]. The stimulating electrode, connected to a battery (Stimulus Isolator 385; WPI) delivered a supramaximal stimulus of 0.75 mA to the nerve, evoking CAPs. The recording electrode was connected to an EPC 9 amplifier (HEKA Elektronik). The signal was amplified 500 times, filtered at 30 kHz, and acquired at 20 kHz. Before recording, optic nerves were equilibrated for at least 30 min in the chamber. The CAP, elicited by the maximum stimulation of 0.75 mA, was recorded at baseline stimulation frequency at 0.1 Hz. During high frequency stimulation, nerves were stimulated by pulses of increasing frequency (1, 4, 7, 15, 25, 50, 75, 100 Hz) each applied for 30 s (ramp protocol).

### Imaging

Live imaging of optic nerves was performed as previously described [8] using an upright confocal laser scanning microscope (Zeiss LSM 510 META/NLO) equipped with an argon laser and a 63× objective (Zeiss 63× IR-Achroplan 0.9 W). The objective was immersed into the aCSF superfusing the optic nerve. Theoretical optical sections of 1.7 μm over a total scanned area of 66.7 μm × 66.7 μm (512 × 512 pixels) of the optic nerve were obtained every 10.4 s in 3 channels, referred as CFP (excitation 458 nm; emission 470–500 nm), FRET (excitation 458 nm; emission long pass 530 nm) and YFP (excitation 514 nm; emission long pass 530 nm).

### FLIM of optic nerves

Optic nerves were excised as described above and transferred in a superfusion chamber where temperature could be maintained at 37°C. Both nerves were kept connected to the chiasm and pinned at the proximal (retina) and distal (chiasm) ends in the incubation chamber (ALA Scientific) using 3% agarose prepared in aCSF. Nerves were constantly superfused with carbogen-bubbled aCSF, and live FLIM of the ATeam1.03^YEMK^, expressed in ThyAT/*Plp*^null/y^ and ThyAT/*Plp*^wt/y^ (control) nerves, was performed using a 2-photon laser scanning microscope (LaVision BioTec) equipped with a Chameleon Vision II pulsed (80 MHz) laser, a 20× water-immersion objective (Zeiss 20×/1.0 W Plan-Apochromat DIC Corr UV-VIS-IR) and a FLIM X1 system with 1 GaAsP (Hybrid) PMT detector (LaVision BioTec). The FLIM X1 had a nominal time-bin window of 27 ps and a dead time of 5.5 ns, and it is used for time-correlated single photon counting (TCSPC) analysis of fluorescence lifetime of the mseCFP (donor of ATeam1.03^YEMK^). The ATeam1.03^YEMK^ donor was excited at 840 nm, and emitted fluorescence was recorded below 495 nm (495-beamsplitter, AHF) in TCSPC mode. The TCSPC data were binned 4 times, and Z-stacks over a volume of 10–16 μm tissue depth, covering an area of approximately 491 × 196 μm, were acquired at a pixel size of 0.192 μm and pixel dwell time of 9.56 μs using a 3D-stitching function of the built-in software ImSpector-Pro 3.60 (LaVision BioTec). Measurements of the instrument response function (IRF) were performed using the same acquisition settings described above (840 nm excitation and photon detection between 400 and 440 nm) by recording second harmonic generation signals from sugar crystals (no immersion). IRF data were used to temporally deconvolve the TCSPC data as described below.

### Solutions

Optic nerves were superfused by aCSF containing 124 mM NaCl, 3 mM KCl, 2 mM CaCl_2_, 2 mM MgSO_4_, 1.25 mM NaH_2_PO_4_, 23 mM NaHCO_3_ and 10 mM glucose, which was continuously bubbled with carbogen. For GD, glucose was removed from the aCSF and substituted by sucrose (Merck Millipore) to maintain the correct osmolarity. For the combination of mitochondrial blockage (MB) with GD (i.e., MB+GD), glucose was substituted by sucrose, and 5 mM sodium azide (Merck Millipore) was added. For high frequency stimulation using the ramp protocol, glucose concentration was reduced to 3.3 mM. All aCSF-based solutions were adjusted for the same pH, sodium concentration, and osmolarity.

### FLIM measurements in HEK293 cells

To correlate the ATeam1.03^YEMK^ sensor FLIM signal with different ATP concentrations, HEK293 cells were transfected with ATeam1.03^YEMK^ using Lipofectamine 2000 (Invitrogen). Transfected cells were kept at 36.5°C during imaging and constantly perfused with an external solution composed of 92 mM NaCl, 18 mM NaHCO_3_, 20 mM HEPES, 5.4 mM KCl, 0.8 mM MgSO_4_, 0.9 mM NaH_2_PO_4_, 0.2 mM Na_2_HPO_4_, 1.8 mM CaCl_2_, and 10 mM glucose. The pipette solution contained 100 mM KCl, 25 mM NMDG, 10 mM BAPTA-4K, 0.3 mM NaN_3_, 10 mM HEPES, and ATP in variable concentrations (0, 1, 2, 4, 6, 10, 24 mM). The pH of the external and internal solution was adjusted to 7.40 and to 7.35, respectively. Cells were patched using a patch pipette with low resistance (1.2–1.5 MΩ), and electrical recordings were used to confirm cell viability and stability via an EPC 9 amplifier controlled by the PatchMaster software (HEKA Elektronik). To confirm cytoplasm access, the pipette solution was supplemented with 2 μM ATTO594, and its fluorescence was monitored at 590–650 nm. Only cells that showed a complete filling (approximately 1–5 min following patch) were considered for further analysis. To acquire the ATeam1.03^YEMK^ FLIM signal, the same acquisition settings as for optic nerves were used, in combination with a time series consisting of 5.5-s time intervals.

### Control experiments for autofluorescence and pH dependency

To estimate the contribution of tissue autofluorescence to the ATP sensor signal obtained by confocal imaging as well as FLIM, optic nerves from wild-type mice lacking the expression of the ATP sensor were imaged using exactly the same imaging conditions and settings as described for optic nerves expressing the ATP sensor. For confocal imaging the autofluorescence signal amounted to 18.0% ± 8.7%, 1.4% ± 0.7%, and 7.9% ± 3.0% for the CFP, FRET, and YFP channel, respectively, and did not change during application of MB+GD (S4 Fig). In the 2-photon FLIM setting, the autofluorescence signal observed in nerves lacking ATP sensor expression was so low that no decay was present and lifetimes could not be calculated (S4 Fig). These data strongly suggest that autofluorescence does not substantially contribute to changes in ATP sensor signal both in confocal imaging and 2-photon FLIM.

While several studies reported that the intensity-based signal of the ATP sensor used in our transgenic mice is almost insensitive to pH changes in the physiological range [8,22,28,29], no information on the pH sensitivity of the fluorescence lifetime of this sensor is available so far. Therefore, HEK293 cells were permeabilized for cations including protons using nigericin (50 μM) and gramicidin (10 μM) in an intracellular buffer consisting of 130 mM KCl, 1.25 mM MgSO_4_, 10 mM NaCl, 10 mM HEPES, and 5 mM glucose (pH 7.4) [44,45]. After permeabilization for 10 min, the intracellular pH was shifted by applying the same buffer titrated to pH 6.8 or pH 8.0. pH shifts were confirmed using the pH indicator dye SNARF-5F imaged by epifluorescence microscopy (Axio Observer Z1; Zeiss, Jena, Germany; Plan-Apochromat 20×/0.8 objective; Axiocam 506 camera with 688 × 552 pixels, 4 × 4 binning, pixel size 0.91 μm × 0.91 μm; excitation 550/25 nm, beam splitter 570 nm, emission 605/70 nm). To assess the fluorescence lifetime of the ATP sensor at these pH values, HEK293 cells were transiently transfected using Lipofectamine 2000 (Invitrogen) with AT1.03^R122K/R126K^, a mutated version of the ATP sensor that contains the same fluorophores and the same ATP binding protein, but does not bind ATP due to 2 point mutations within the ATP binding domain [22]. This ATP-binding-deficient version of the ATP sensor was used instead of the intact sensor expressed in the ThyAT mouse line because, in the latter, changes of the fluorescence lifetime could be caused either by a direct effect of pH on the fluorophores or by changes in the ATP concentration due to active, energy-consuming pH regulation of the cells. Cells were permeabilized as described and imaged using the same FLIM conditions as for optic nerves.

### Data analysis

Data analysis for CAP and ATP sensor signal was done as previously described [8].

#### CAP

Optic nerve function was monitored quantitatively as the area under the supramaximal CAP. The CAP area is proportional to the total number of excited axons and represents a convenient and reliable means of monitoring optic nerve axon function [7,43]. CAP was expressed as area under the CAP in a range of approximately 1.5 ms, defined by the beginning of the response (typically at 0.2 ms after the stimulus) and by the lowest point that marked the end of the second peak of the curve characteristic of the optic nerve evoked CAP. Data were normalized to the mean obtained during the initial 15 min (defined as baseline, i.e., no experimental condition applied). Results from several nerves were pooled, averaged, and plotted against time.

#### ATP

The relative amount of ATP in the optic nerve was calculated in 2 steps. Initially, the mean intensity of FRET and CFP channel signal was extracted from the imaging time series (in Fiji), and the F/C ratio was calculated. All fibers visible in the field of view were included in the analysis without any prior selection. The F/C ratio was then normalized to 0 using the F/C value obtained by application of MB+GD at the end of each experiment, i.e., by depleting all axons from ATP. Furthermore, F/C values obtained during baseline recordings at the beginning of each experiment were set to 1, as described previously [8].

#### ATP and CAP parameters

For GD, MB, and MB+GD conditions, *m* coefficients for the slope intercepts at the point of maximal change of the signal were used to calculate the CAP and ATP rate per second, during the initial decay and the recovery following reperfusion with aCSF containing 10 mM glucose. The time of the start of decay/recovery of the ATP and CAP curves was defined as the first time point at which the slope intercept of the signal was above a threshold value based on the standard deviation calculated at the baseline. For analysis of high frequency stimulation, the overall amplitude obtained by averaging the data at the time points within the last 15 s of the stimulation was considered for both CAP and ATP.

### FLIM analysis

The TCSPC raw data were stitched in ImSpector and exported as tif files for further processing into MATLAB analysis routine. Bi-exponential and multi-exponential decays were used to analyze data obtained in HEK293 cells and optic nerves, respectively. In time-domain fluorescence decay measurements, the measured histogram *f*(*t*) is a convolution of exponential decay functions with the IRF, *h*(*t*):

$$f(t)=\int_{-\infty }^{t}dt^{\prime }\sum_{j=1}^{m}\frac{A_{j}}{\tau_{j}}exp\left(-\frac{t-t^{\prime }}{\tau_{j}}\right)h(t^{\prime })$$

The reported lifetime values were then calculated as average fluorescence lifetime $\bar{\tau }$, defined as

$$\bar{\tau }=\frac{\sum_{j=1}^{m}A_{j}\tau_{j}}{\sum_{j=1}^{m}A_{j}}$$

obtained by integrating *f*(*t*)*t* over time *t*

$$F_{1}=\left(\int_{-\infty }^{\infty }dt^{\prime }t^{\prime }h(t^{\prime })\right)\sum_{j=1}^{m}A_{j}+\left(\int_{-\infty }^{\infty }dt^{\prime }h(t^{\prime })\right)\sum_{j=1}^{m}A_{j}\tau_{j}$$

where integrals over *h*(*t*) are the 0th and first moment of the IRF, respectively:

$$H_{0}=\int_{-\infty }^{\infty }dt^{\prime }h(t^{\prime })\;and\;H_{1}=\int_{-\infty }^{\infty }dt^{\prime }t\prime h(t^{\prime })$$

And, in an analogous way, by integrating *f*(*t*) over time:

$$F_{0}=\int_{-\infty }^{\infty }dt^{\prime }f(t^{\prime })$$

We can then derive

$$\bar{\tau }=\frac{F_{1}}{F_{0}}-\frac{H_{1}}{H_{0}}$$

Transformation of the pixel-wise TCSPC histogram *i*(*t*_*k*_) data into the phasor coordinates *g* and *s* is calculated as

$$g=\frac{\sum_{k=1}^{n}i(t_{k})\cos\left[2\pi f_{r}(t_{k}-\delta_{\mathrm{IRF}})\right]}{\sum_{k=1}^{n}i(t_{k})}$$

$$s=\frac{\sum_{k=1}^{n}i(t_{k})\sin\left[2\pi f_{r}(t_{k}-\delta_{IRF})\right]}{\sum_{k=1}^{n}i(t_{k})}$$

Here, *i*(*t*_*k*_) is the number of counts in the histogram bin *t*_*k*_, *f*_r_ is the repetition frequency of the excitation laser, and δ_IRF_ is the average time of the IRF. For an ideal mono-exponential decay with lifetime τ the expected coordinates are

$$g=\frac{1}{1+{\left(2\pi f_{r}\tau \right)}^{2}}$$

$$s=\frac{2\pi f_{r}\tau }{1+{\left(2\pi f_{r}\tau \right)}^{2}}$$

Plotting the coordinates *s* versus *g* for all pixels in the FLIM image allows one to analyze the lifetime distribution without fitting a model function to the TCSPC data. For τ = 0, one finds *g* = 1 and *s* = 0, and for long lifetimes, *g* = 0 and *s* = 0. A maximum of *s* = 0.5 is found for τ = 1/2π*f*_*r*_. Generally, all possible coordinates lie within the semicircle with radius 0.25 and center (0.5, 0). A clockwise shift of the distributions between 2 FLIM recordings indicates a decrease of lifetimes, and vice versa.

For analysis of the variability of the ATP sensor fluorescence lifetime within axons, axons were segmented manually using Fiji [46]. The variability of the ATP sensor signal within an axon was assessed by calculation of the CV of the fluorescence lifetime of all pixels within this axon. To address the variability of the ATP sensor signal across different axons within a nerve, first the mean fluorescence lifetime of all pixels within each axon, and then the CV of these mean axonal fluorescence lifetimes of all axons within this nerve, was calculated. For the analysis of *Plp*^null/wt^ mice, care was taken to segment axonal segments of 20–25 μm length, which is much shorter than the length of internodes in the optic nerve [47–50]. It follows that most of the axonal segments analyzed will be covered by a single internode of myelin.

### Quantitative RT-PCR

RNA was isolated from the corpora callosa of 10-week-old male mice of the genotypes indicated, and quantitative RT-PCR performed as described previously [32]. *Ppia* and *Rps13*, which were not different between genotypes, were used as standards. Primers were specific for *Glut1* (forward 5′-TTCTCTGTCGGCCTCTTTGT, reverse 5′-TCAAAGGACTTGCCCAGTTT), *Mct1* (forward 5′-ATGCTGCCCTGTCCTCCT, reverse 5′-CCACAAGCCCAGTACGTGTAT), *Ppia* (forward 5′-CAC AAACGGTTCCCAGTTTT, reverse 5′-TTCCCAAAGACCACATGCTT), and *Rps13* (forward 5′-CGAAAGCACCTTGAGAGGAA, reverse 5′-TTCCAATTAGGTGGGAGCAC). Statistical analysis was performed in GraphPad Prism 6.0 using the 2-tailed *t* test assuming equal mean square error according to a previously performed *F*-test.

### Immunoblot and quantification

#### Brain myelin preparation

Male C57Bl6/N-*Plp*^null/y^ (*Plp*^null/y^) mice and their corresponding wild-type male littermates were sacrificed at the age of 75 days. A light weighted membrane fraction enriched in myelin was obtained from frozen half brains by sucrose density gradient centrifugation essentially as described previously [51]. Briefly, after homogenizing the brains in 0.32 M sucrose solution containing protease (cOmplete, Roche) and deacetylase inhibitors (10 mM nicotinamide, 0.5 μM Trichostatin A, Sigma), a first fraction enriched in myelin was obtained by density gradient centrifugation on a 0.85 M sucrose cushion. After washing and 2 consecutive osmotic shocks, the final brain myelin fraction was purified by sucrose gradient centrifugation as before. The myelin fraction was washed and suspended in TBS buffer (137 mM NaCl, 20 mM Tris/HCl [pH 7.4], 4°C) supplemented with protease (cOmplete, Roche) and deacetylase inhibitors (10 mM nicotinamide, 0.5 μM Trichostatin A, Sigma). Protein concentration was determined using the DC Protein Assay (Bio-Rad) according to the manufacturer’s instructions.

#### Immunoblotting

Immunoblotting was performed as described before [52], with some modifications. Briefly, 3 to 20 μg of myelin protein sample in sample buffer containing 2% (w/v) LDS, 0.5% (v/v) Triton X-100, and 0.5% (w/v) sodium deoxycholate was separated on 12% polyacrylamide-SDS gels in Laemmli buffer. For immunoblotting, proteins were blotted onto low fluorescent polyvinylidene difluoride membranes (Immobilon-FL PVDF, IPFL00010, Merck Millipore). For quantification of total protein, blots were stained by fast green (5 mg/l fast green, Sigma, in 6.7% [v/v] acetic acid and 30% [v/v] methanol) immediately after transfer for 5 min, washed twice for 30 s in 6.7% (v/v) acetic acid and 30% (v/v) methanol, and imaged using the 700-nm channel of a near-infrared scanned (Odyssey, Licor). Consecutively, blots were destained in 50% (v/v) ethanol in TBS (150 mM NaCl, 10 mM Tris/HCl [pH 7.4]) and blocked with 5% (w/v) skim milk in TBS. Primary antibodies were diluted in 5% (w/v) skim milk in TBST (150 mM NaCl, 10 mM Tris/HCl [pH 7.4], 0.5% [v/v] Tween 20), and incubation was for 48 h at 4°C. Primary antibodies were specific for GLUT1 (1:1,000) [53], MCT1 (1:1,000) [42], β-actin (ACTB; 1:1,000, MAB1501, Merck Millipore), and PLP/DM20 (1:5,000, A431) [54]. After washing with TBST, blots were incubated with near-infrared fluorophore-coupled secondary antibodies diluted in 5% (w/v) skim milk in TBST (1:10,000, Mouse IgG [H&L] Antibody DyLight 680 Conjugated, 610-144-002; Rabbit IgG [H&L] Antibody DyLight 800 Conjugated, 611-145-002; Rockland), washed with TBST, washed finally with TBS, and detected using a near-infrared fluorescence scanner (Odyssey, Licor). For quantification of GLUT1 and MCT1 abundance, images obtained from the near-infrared fluorescence imager were analyzed using the Image Studio software (Licor). Signal intensities were normalized to their corresponding total protein load as quantified by fast green staining. Normalized signal intensities for GLUT1 and MCT1 obtained for myelin samples of *Plp*^null/y^ mice were normalized to the mean of the respective signal intensities of myelin samples from wild-type mice.

### Electron microscopy

#### Sample preparation for transmission electron microscopy (TEM)

Mice were sacrificed by cervical dislocation, and their optic nerves carefully removed. For conventional preparation or immunoelectron microscopy, nerves were fixed by immersion fixation in phosphate-buffered fixing solution containing 4% formaldehyde and 2.5% glutaraldehyde or 4% formaldehyde and 0.25% glutaraldehyde, respectively. Nerves fixed with the higher concentration of glutaraldehyde were processed for imaging by FIB-SEM [1]; the mildly fixed nerves were prepared for ultrathin cryosectioning and immunolabeling (described in detail in [55]). Ultrathin cryosections were labeled with antibodies raised in rabbit directed against GLUT1 and MCT1 (GLUT1: 1:200 [53]; MCT1: 1:100 [42]) and protein A gold with a particle size of 10 nm (CMC Utrecht, the Netherlands). Images were taken with a LEO 912 electron microscope (Carl Zeiss Microscopy, Oberkochen, Germany).

For preparation by high-pressure freezing, the freshly prepared nerves were cut in half and processed as described in detail previously [55]. The stained sections were analyzed using a LEO 912 AB electron microscope (Zeiss, Oberkochen, Germany) equipped with a CCD camera (TRS, Moorenweis, Germany). For quantification of axonal pathologies and myelin alterations, 4 × 4 tile images with 10% overlap corresponding to approximately 276 μm^2^ were acquired. Imaging areas were selected for good tissue preservation but otherwise randomly chosen. Per mouse, all axons within 15 images (2,565 to 5,036 axons per mouse) were quantified with respect to presence of (i) compact myelin, (ii) axonal swellings, and (iii) cytosol-filled non-compacted myelin areas breaching otherwise compact myelin sheaths (cytosolic channels) using the cell counter plug-in in ImageJ.

#### Sample preparation for FIB-SEM

Samples were prepared as described before [20,56], with some modifications. Mice were sacrificed by cervical dislocation, and their optic nerves carefully removed. The nerves were cut in half and placed in the fixative (4% formaldehyde [Serva] and 2.5% glutaraldehyde [Science Services] in 0.1 M PB [109.5 mM NaH_2_PO_4_•H_2_O, 93.75 mM Na_2_HPO_4_•2H_2_O, and 86.2 mM NaCl]). The samples were further processed following OTO protocol [57]. The samples were washed in 0.1 M PB (3 × 15 min) and then incubated for 3 h at 4°C in 2% OsO_4_ (Electron Microscopy Sciences) and 0.25% K_4_[Fe(CN)_6_] (Electron Microscopy Sciences). After washing with H_2_O (3 × 15 min), the samples were incubated with 0.1% thiocarbohydrazide (Sigma-Aldrich) for 1 h at room temperature. For further contrast enhancement, the tissue was treated with 2% OsO_4_ for 90 min. The samples were washed with H_2_O (3 × 15 min) and contrasted overnight with 2% uranyl acetate (SPI-Chem) at 4°C. The samples were washed again with H_2_O (3 × 15 min), followed by dehydration in an increasing acetone series (30%, 50%, 75%, 90%, 3 × 100%). The tissue was infiltrated with increasing concentrations of Durcupan (Sigma-Aldrich, components A, B, C) for 2 h each (25%, 50%, 75% Durcupan in acetone) and then incubated in 100% Durcupan overnight. Fresh Durcupan with accelerator (component D) was added to the samples for 5 h, before the samples were embedded in resin blocks. They were polymerized for 48 h at 60°C.

#### FIB-SEM

The samples were processed as previously described [56]. They were trimmed with a 90° diamond trimming knife (Diatome, Biel, Switzerland) and attached to the SEM stub (Science Services, Pin 12.7 mm × 3.1 mm) by a silver-filled epoxy (Epoxy Conductive Adhesive, EPO-TEK EE 129–4; Electron Microscopy Sciences) and polymerized at 60°C overnight. Following polymerization the samples were coated with a 10-nm gold layer using the sputter coater EM ACE600 (Leica) at 35 mA current and placed into the Crossbeam 540 focused ion beam scanning electron microscope (Carl Zeiss Microscopy). To protect the surface and ensure even milling, a 400-nm platinum layer was deposited on top of the region of interest. Atlas 3D software (Atlas 5.1, Fibics, Canada) was used to collect the 3D data. Samples were exposed with a 15-nA current, and a 7-nA current was used to polish the surface. The images were acquired at 1.5 kV with the ESB detector (450 V ESB grid, 600 pA, pixel size *x/y* 5 nm) in a continuous mill and acquire mode using 1.5 nA for the milling aperture (*z*-step 25 nm).

#### Image post-processing

The following post-processing steps were performed using Fiji [46]. The image stack was cropped and inverted, and the SIFT algorithm was used to align any translational shift. The images were smoothed, a local contrast enhancement was applied (CLAHE: blocksize 127, histogram bins 55, maximum slope 1.5), and a Gaussian blur (sigma 1) and a sharpening were performed. The 3D reconstruction was done manually using IMOD [58] and MIB [59].

### Presentation of data

Data are presented as mean ± SEM or as bar graphs showing the mean (bar), SEM (error bars), and the single data points (dots). Data consisting of 2 groups were statistically evaluated using Student *t* test or Welch’s test as indicated in the figure legends. Data consisting of 3 groups were evaluated using ANOVA with Tukey’s post hoc test or ANOVA on ranks with Dunn’s post hoc test as indicated in the figure legends. Statistically significant differences are illustrated: *p < 0.05; **p < 0.01; ***p < 0.001.

## Supporting information

S1 Data Numerical values that were used to generate graphs of all figures and supplementary figures in Excel (xlsx) format. (XLSX)

S1 Fig Two-photon fluorescence lifetime imaging of the ATP sensor ATeam1.03^YEMK^ in optic nerves. (A) Intensity of the ATP sensor signal, color-coded as shown in the inset. Scale bar: 50 μm. (B) F/C ratio of the ATP sensor, which is indicative of the ATP concentration, color-coded as shown in the inset. Scale bar: 50 μm. (C) Fluorescence lifetime of the ATP sensor in a *Plp*^wt/y^ (left) and a *Plp*^null/y^ (right) optic nerve, color-coded as shown in the inset. Scale bar: 50 μm. The image in the circle shows a magnification of the encircled area, highlighting the differences in fluorescence lifetime in axonal swellings.
(TIF)

S2 Fig Calibration of the ATP sensor ATeam1.03^YEMK^ in HEK293 cells. HEK293 cells were patch-clamped using intracellular pipette solutions with different concentrations of ATP and imaged using 2-photon FLIM and the same imaging conditions as described for the optic nerves. A clear and direct dependency of fluorescence lifetime on ATP concentration was observed. *n* = 5, 3, 4, 2, 3, 2, and 2 cells from low to high concentration of ATP. Shown is the mean ± SEM. Data underlying this figure can be found in S1 Data.
(TIF)

S3 Fig Phasor analysis of the fluorescence lifetime. (A) Phasor analysis showing an increase in the fluorescence lifetime in the *Plp*^null/y^ axons as indicated by a left shift along the *g* (mcosɸ) axis. (B) Phasor analysis indicating the maximum shift along the *g* (mcosɸ) axis of the fluorescence decay in the axons during MB+GD. The phasor analysis was performed on the same set of optic nerves as in Fig 2.
(TIF)

S4 Fig Technical controls for ATP imaging. (A and B) To address the contribution of tissue autofluorescence (autofl.) to the signal of the ATP sensor in confocal microscopy, wild-type nerves lacking ATP sensor expression (*n* = 4 optic nerves) were imaged using the same imaging conditions used for imaging the ATP sensor in optic nerves of ThyAT mice. The signal is much lower for all 3 imaging channels (A; normalized to the mean basal fluorescence signal in each channel observed in optic nerves expressing the ATP sensor) and is unchanged during MB+GD (B, normalized to the fluorescence prior to MB+GD). (C) Imaging of wild-type nerves (*n* = 3) using FLIM and the same settings used for imaging the ATP sensor did not result in any signal, thereby excluding a contribution of tissue autofluorescence to the fluorescence lifetime measurements of the ATP sensor. The inset shows the instrument response function (IRF). (D and E) To study the pH dependency of the fluorescence lifetime of the ATP sensor, HEK293 cells were permeabilized for protons using nigericin and gramicidin and incubated in solutions with different pH. The intracellular pH is shifted accordingly, as monitored by the pH-sensitive dye SNARF-5F (D; data normalized to the SNARF-5F signal at pH 7.4; *n* = 4 and 3 experiments with a total of 399 and 299 cells for pH 6.8 and pH 8.0, respectively). When the intracellular pH of HEK293 cells expressing the ATP-binding-deficient version of the ATP sensor, AT1.03^R122K/R126K^, was modulated accordingly, the fluorescence lifetime of the ATP sensor was slightly affected (E; *n* = 6 experiments corresponding to the mean of 27 to 42 cells per experiment; ***p < 0.001, ANOVA repeated measurements, Tukey’s post hoc test). (F) During MB+GD the signal in the YFP channel remains almost invariable and shows no difference between control (Ctrl) and *Plp*^null/y^ mice. *n* = 7 and 4 optic nerves from *N* = 7 and 4 mice for Ctrl and *Plp*^null/y^ mice, respectively. *p >* 0.05, Student *t* test. Data underlying this figure can be found in S1 Data.
(TIF)

S5 Fig Different types of analysis reveal the same mean fluorescence lifetime. Both the analysis used for calculation of the coefficient of variation of the fluorescence lifetime across pixels in an individual axon [Ax (single)] and across axons in an individual nerve [Ax (nerve)] reveal the same mean fluorescence lifetime as the analysis of the whole optic nerve [ON (whole); same data as in Fig 2D], suggesting that the subsampling of manually segmented axons provides a representative set of axons; 88 and 79 axons from *n* = 9 and 8 nerves each from *N* = 5 and 4 animals each were analyzed for control and *Plp*^null/y^ nerves, respectively. n.s.: *p >* 0.05, ANOVA, Tukey’s post hoc test. Data underlying this figure can be found in S1 Data.
(TIF)

S6 Fig Analysis of ATP levels in *Plp*^null/wt^ mice. In *Plp*^null/wt^ mice each oligodendrocyte inactivates either the wild-type or the mutant allele of the X-chromosomal *Plp* gene, leading to a mosaic expression of PLP. (A) The fluorescence lifetime of the ATP sensor in axons of optic nerves from *Plp*^null/wt^ mice (i.e., heterozygous for *Plp*) is intermediate between that of control and *Plp*^null/y^ (i.e., *Plp* knockout) mice, indicative of an intermediate mean basal concentration of ATP. Data of control (Ctrl) and *Plp*^null/y^ mice are the same as in Fig 2D and are shown here for better comparison only. *n* = 7 optic nerves from *N* = 4 mice for *Plp*^null/wt^ mice. **p < 0.01, ***p < 0.001; ANOVA, Tukey’s post hoc test. (B) The coefficient of variation (CV) of the ATP sensor signal also provides evidence of an intermediate phenotype of *Plp*^null/wt^ mice; 136 and 229 stretches of axons (each 20–25 μm long) from 3 and 6 optic nerves were included in this analysis for Ctrl and *Plp*^null/wt^ mice, respectively. Data for *Plp*^null/y^ mice are the same as in Fig 2F and are shown here for better comparison only. *p < 0.05, **p < 0.01, ***p < 0.001; ANOVA on ranks, Dunn’s post hoc test. Data underlying this figure can be found in S1 Data.
(TIF)

S1 Movie Movie showing the 3D reconstruction of a cytoplasmic channel in a *Plp*^null/y^ optic nerve. The 3D dataset was acquired with a focused ion beam scanning electron microscope. MIB and IMOD were used to reconstruct different parts of an axon manually. Abaxonal myelin (blue), inner myelin with cytoplasmic channels (yellow), axon (light blue), and organelle-like structures in between the myelin sheaths (purple) are shown. Scale bar: 500 nm.
(MP4)

S1 Raw images Annotated raw images of the Western blots shown in Fig 6. Original, uncropped, and minimally adjusted images of the Western blot data shown in Fig 6.
(PDF)

## Abbreviations

aCSF: artificial cerebrospinal fluid
CAP: compound action potential
CNS: central nervous system
CV: coefficient of variation
FIB-SEM: focused ion beam scanning electron microscopy
FLIM: fluorescence lifetime imaging
GD: glucose deprivation
IRF: instrument response function
MB: mitochondrial blockage
SPG2: spastic paraplegia type 2
TCSPC: time-correlated single photon counting
