# Supplementary figures and images for: Structural myelin defects are associated with low axonal ATP levels but rapid recovery from energy deprivation in a mouse model of spastic paraplegia
Source: PLoS Biol. 2020 Nov 16;18(11):e3000943. doi: 10.1371/journal.pbio.3000943 (PMC7704050; doi:10.1371/journal.pbio.3000943)

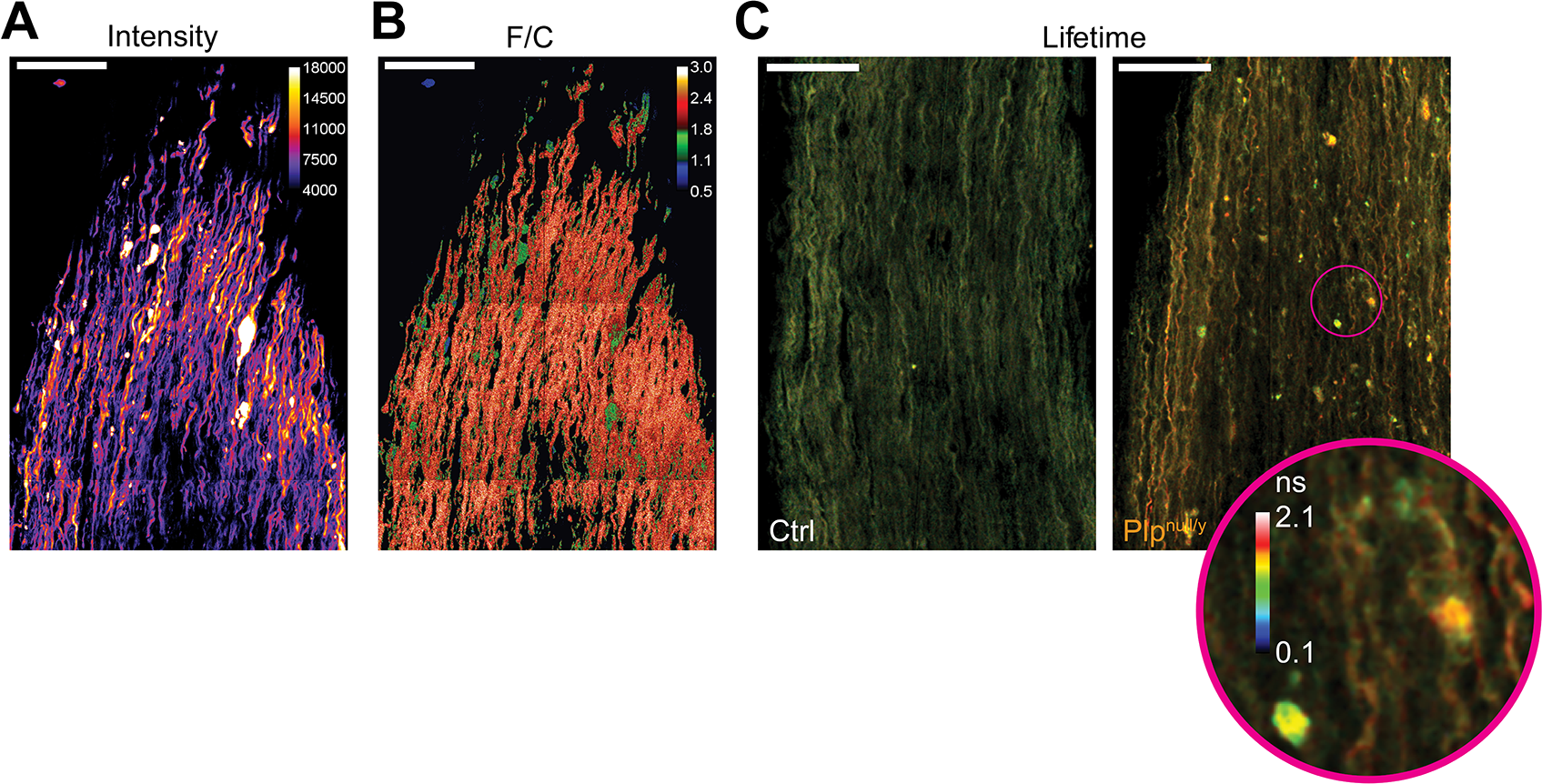

Supplement: S1 Fig — (A) Intensity of the ATP sensor signal, color-coded as shown in the inset. Scale bar: 50 μm. (B) F/C ratio of the ATP sensor, which is indicative of the ATP concentration, color-coded as shown in the inset. Scale bar: 50 μm. (C) Fluorescence lifetime of the ATP sensor in a Plpwt/y (left) and a Plpnull/y (right) optic nerve, color-coded as shown in the inset. Scale bar: 50 μm. The image in the circle shows a magnification of the encircled area, highlighting the differences in fluorescence lifetime in axonal swellings. (TIF) [file pbio.3000943.s002.tif]

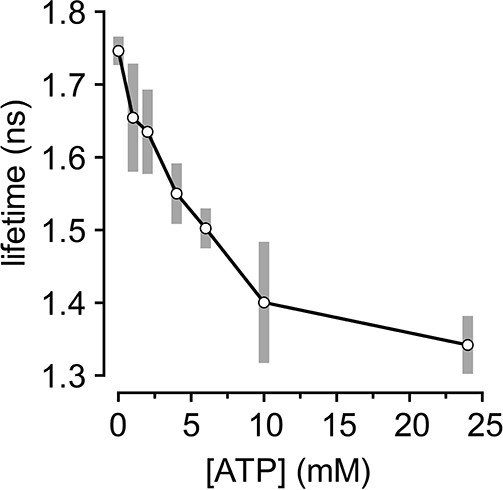

Supplement: S2 Fig — HEK293 cells were patch-clamped using intracellular pipette solutions with different concentrations of ATP and imaged using 2-photon FLIM and the same imaging conditions as described for the optic nerves. A clear and direct dependency of fluorescence lifetime on ATP concentration was observed. n = 5, 3, 4, 2, 3, 2, and 2 cells from low to high concentration of ATP. Shown is the mean ± SEM. Data underlying this figure can be found in S1 Data. (TIF) [file pbio.3000943.s003.tif]

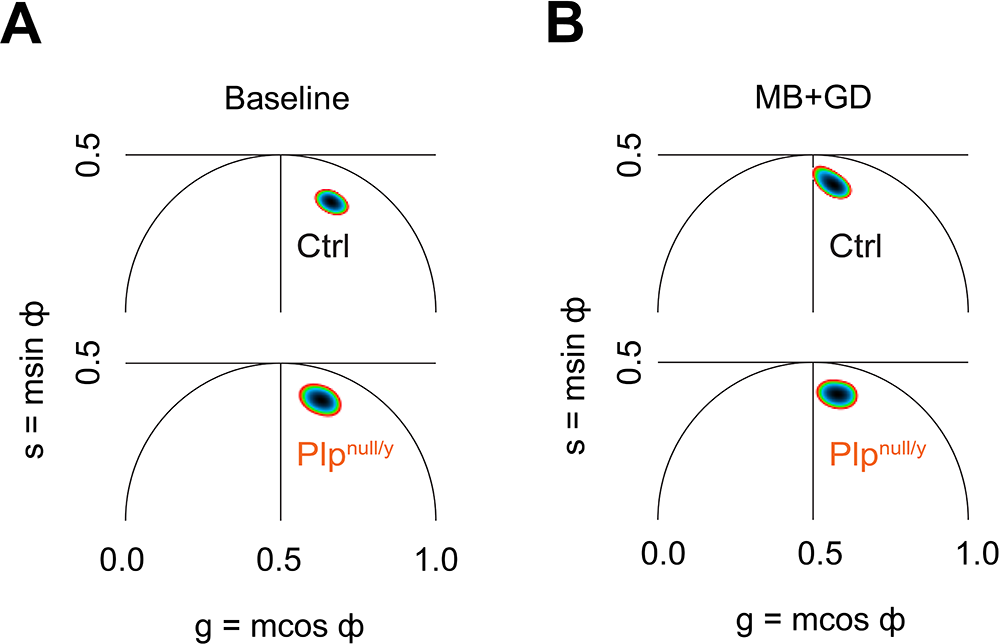

Supplement: S3 Fig — (A) Phasor analysis showing an increase in the fluorescence lifetime in the Plpnull/y axons as indicated by a left shift along the g (mcosɸ) axis. (B) Phasor analysis indicating the maximum shift along the g (mcosɸ) axis of the fluorescence decay in the axons during MB+GD. The phasor analysis was performed on the same set of optic nerves as in Fig 2. (TIF) [file pbio.3000943.s004.tif]

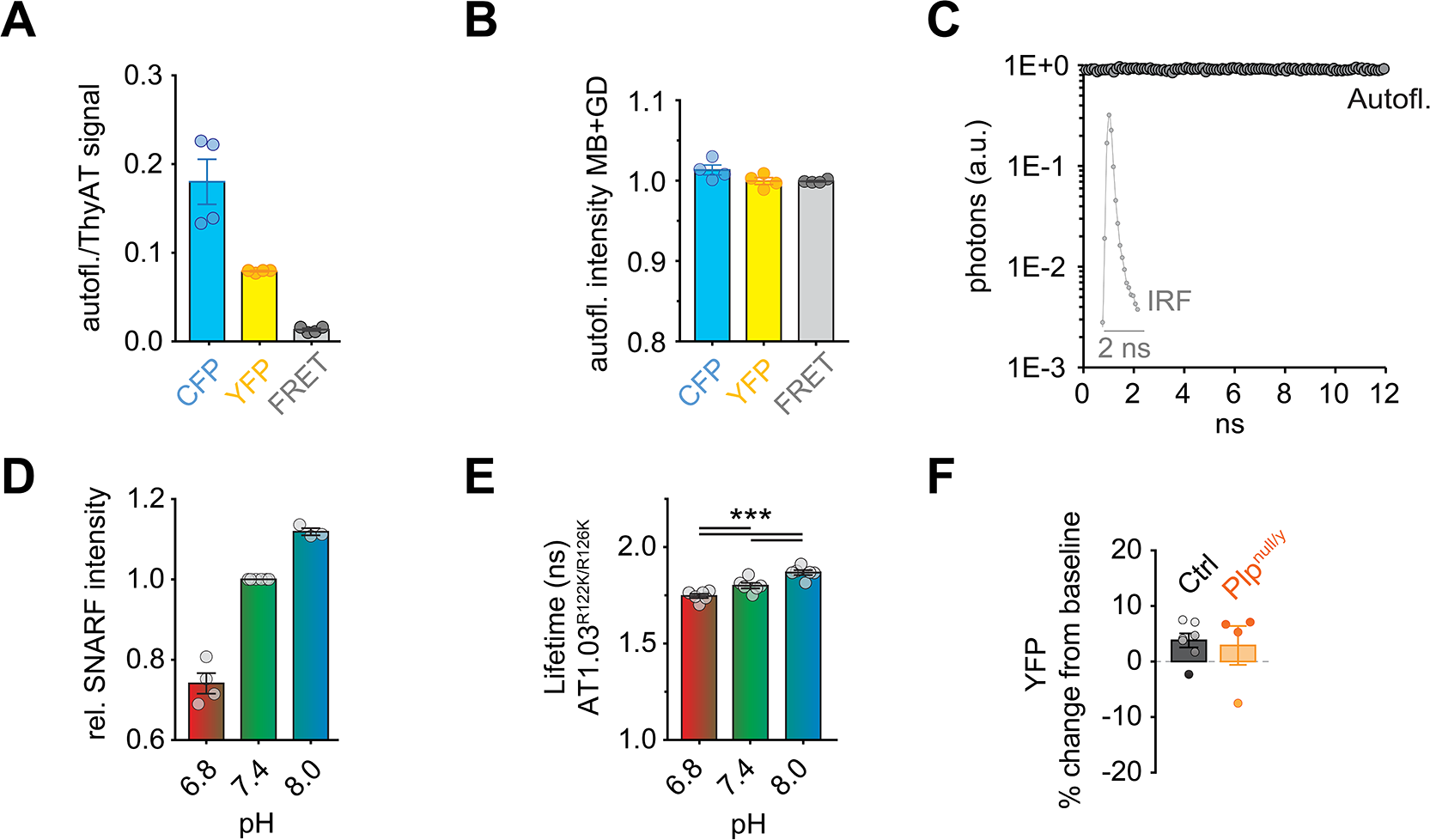

Supplement: S4 Fig — (A and B) To address the contribution of tissue autofluorescence (autofl.) to the signal of the ATP sensor in confocal microscopy, wild-type nerves lacking ATP sensor expression (n = 4 optic nerves) were imaged using the same imaging conditions used for imaging the ATP sensor in optic nerves of ThyAT mice. The signal is much lower for all 3 imaging channels (A; normalized to the mean basal fluorescence signal in each channel observed in optic nerves expressing the ATP sensor) and is unchanged during MB+GD (B, normalized to the fluorescence prior to MB+GD). (C) Imaging of wild-type nerves (n = 3) using FLIM and the same settings used for imaging the ATP sensor did not result in any signal, thereby excluding a contribution of tissue autofluorescence to the fluorescence lifetime measurements of the ATP sensor. The inset shows the instrument response function (IRF). (D and E) To study the pH dependency of the fluorescence lifetime of the ATP sensor, HEK293 cells were permeabilized for protons using nigericin and gramicidin and incubated in solutions with different pH. The intracellular pH is shifted accordingly, as monitored by the pH-sensitive dye SNARF-5F (D; data normalized to the SNARF-5F signal at pH 7.4; n = 4 and 3 experiments with a total of 399 and 299 cells for pH 6.8 and pH 8.0, respectively). When the intracellular pH of HEK293 cells expressing the ATP-binding-deficient version of the ATP sensor, AT1.03R122K/R126K, was modulated accordingly, the fluorescence lifetime of the ATP sensor was slightly affected (E; n = 6 experiments corresponding to the mean of 27 to 42 cells per experiment; ***p < 0.001, ANOVA repeated measurements, Tukey’s post hoc test). (F) During MB+GD the signal in the YFP channel remains almost invariable and shows no difference between control (Ctrl) and Plpnull/y mice. n = 7 and 4 optic nerves from N = 7 and 4 mice for Ctrl and Plpnull/y mice, respectively. p > 0.05, Student t test. Data underlying this figure can be found in S1 Data. (T [file pbio.3000943.s005.tif]

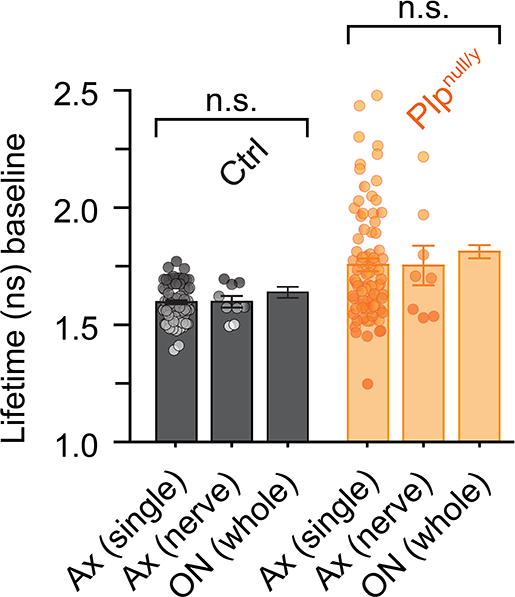

Supplement: S5 Fig — Both the analysis used for calculation of the coefficient of variation of the fluorescence lifetime across pixels in an individual axon [Ax (single)] and across axons in an individual nerve [Ax (nerve)] reveal the same mean fluorescence lifetime as the analysis of the whole optic nerve [ON (whole); same data as in Fig 2D], suggesting that the subsampling of manually segmented axons provides a representative set of axons; 88 and 79 axons from n = 9 and 8 nerves each from N = 5 and 4 animals each were analyzed for control and Plpnull/y nerves, respectively. n.s.: p > 0.05, ANOVA, Tukey’s post hoc test. Data underlying this figure can be found in S1 Data. (TIF) [file pbio.3000943.s006.tif]

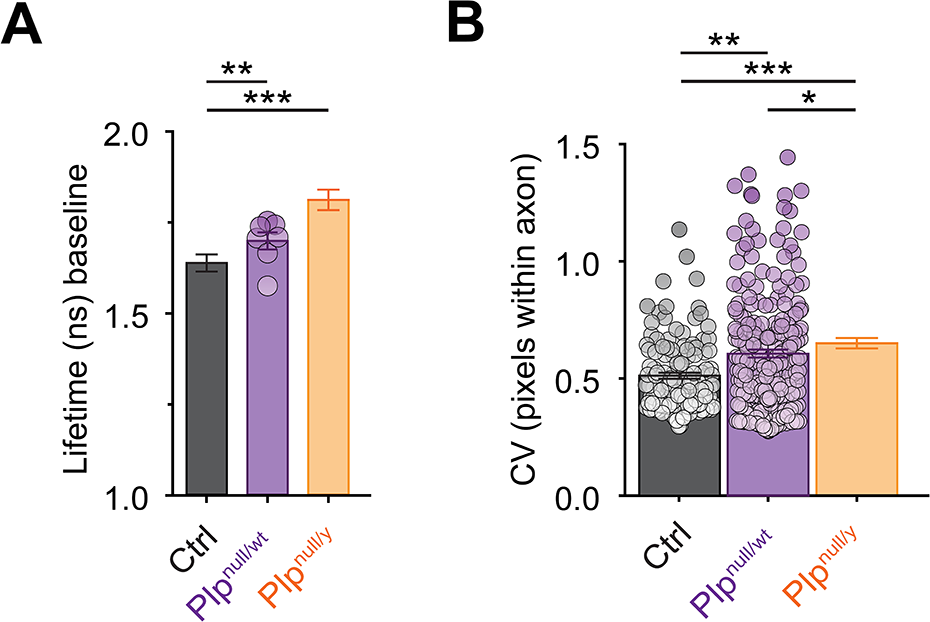

Supplement: S6 Fig — In Plpnull/wt mice each oligodendrocyte inactivates either the wild-type or the mutant allele of the X-chromosomal Plp gene, leading to a mosaic expression of PLP. (A) The fluorescence lifetime of the ATP sensor in axons of optic nerves from Plpnull/wt mice (i.e., heterozygous for Plp) is intermediate between that of control and Plpnull/y (i.e., Plp knockout) mice, indicative of an intermediate mean basal concentration of ATP. Data of control (Ctrl) and Plpnull/y mice are the same as in Fig 2D and are shown here for better comparison only. n = 7 optic nerves from N = 4 mice for Plpnull/wt mice. **p < 0.01, ***p < 0.001; ANOVA, Tukey’s post hoc test. (B) The coefficient of variation (CV) of the ATP sensor signal also provides evidence of an intermediate phenotype of Plpnull/wt mice; 136 and 229 stretches of axons (each 20–25 μm long) from 3 and 6 optic nerves were included in this analysis for Ctrl and Plpnull/wt mice, respectively. Data for Plpnull/y mice are the same as in Fig 2F and are shown here for better comparison only. *p < 0.05, **p < 0.01, ***p < 0.001; ANOVA on ranks, Dunn’s post hoc test. Data underlying this figure can be found in S1 Data. (TIF) [file pbio.3000943.s007.tif]
