# Supplementary material for: Structural myelin defects are associated with low axonal ATP levels but rapid recovery from energy deprivation in a mouse model of spastic paraplegia
Source: PLoS Biol. 2020 Nov 16;18(11):e3000943. doi: 10.1371/journal.pbio.3000943 (PMC7704050; doi:10.1371/journal.pbio.3000943)

S1_raw_images for Trevisol and Kusch *et al.*

raw data for Trevisiol and Kusch et al., figure 6A
primary antibody: rabbit anti-GLUT1
secondary antibody: goat anti-rabbit-DyeLight800
detection at 800nm using a Odyssey Classic Imaging System (Licor)

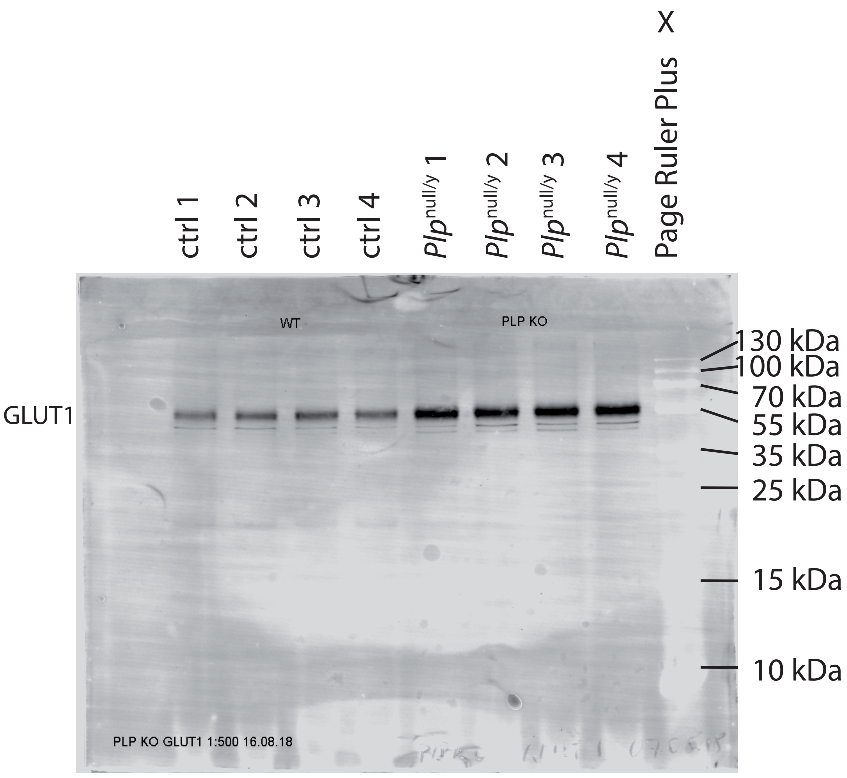

raw data for Trevisiol and Kusch et al., figure 6A
Reprobe of membrane used for GLUT1
primary antibody: rabbit anti-PLP
secondary antibody: goat anti-rabbit-DyeLight700
detection at 800nm using a Odyssey Classic Imaging System (Licor)

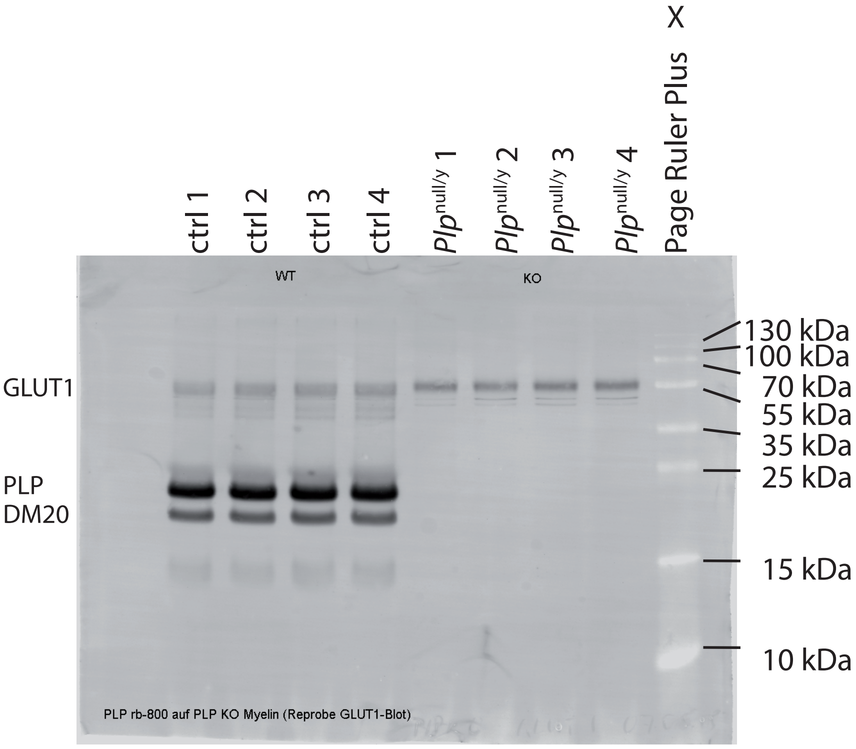

raw data for Trevisiol and Kusch et al., figure 6A
primary antibody: rabbit anti-MCT1
secondary antibody: goat anti-rabbit-DyeLight800
detection at 800nm using a Odyssey Classic Imaging System (Licor)

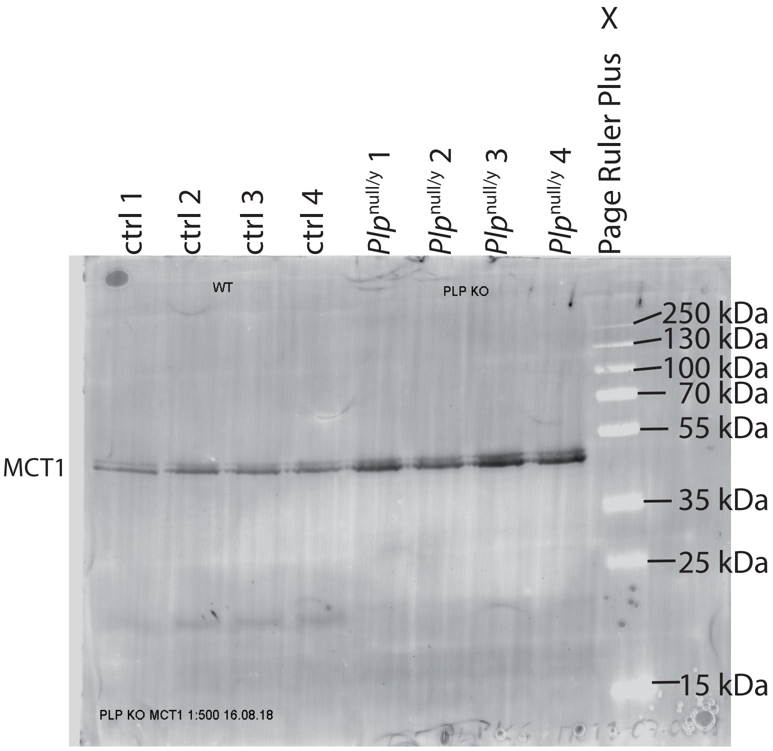

raw data for Trevisiol and Kusch et al., figure 6A
Reprobe of membrane used for MCT1
primary antibody: mouse anti-ACTB
secondary antibody: goat anti-mouse-DyeLight700
detection at 700nm using a Odyssey Classic Imaging System (Licor)

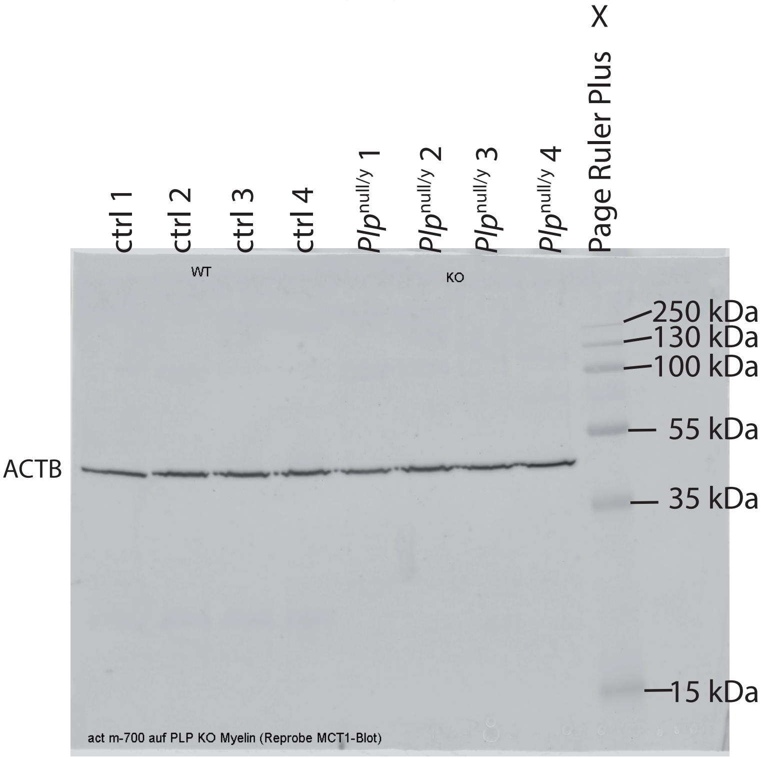

Supplement: S1 Raw images — Original, uncropped, and minimally adjusted images of the Western blot data shown in Fig 6. (PDF) [file pbio.3000943.s009.pdf]
